# Novel antibodies reveal presynaptic localization of C9orf72 protein and reduced protein levels in *C9orf72* mutation carriers

**DOI:** 10.1186/s40478-018-0579-0

**Published:** 2018-08-03

**Authors:** Petra Frick, Chantal Sellier, Ian R. A. Mackenzie, Chieh-Yu Cheng, Julie Tahraoui-Bories, Cecile Martinat, R. Jeroen Pasterkamp, Johannes Prudlo, Dieter Edbauer, Mustapha Oulad-Abdelghani, Regina Feederle, Nicolas Charlet-Berguerand, Manuela Neumann

**Affiliations:** 1 German Center for Neurodegenerative Diseases (DZNE), Otfried-Müllerstr. 23, 72076 Tübingen, Germany; 2 Institut de Génétique et de Biologie Moléculaire et Cellulaire (IGBMC), INSERM U964, CNRS UMR7104, Strasbourg University, 67400 Illkirch, France; 3 Department of Pathology, University of British Columbia and Vancouver General Hospital, Vancouver, Canada; 4 INSERM/UEVE UMR 861, I-STEM, AFM, 91100 Corbeil-Essonnes, France; 5 Department of Translational Neuroscience, Brain Center Rudolf Magnus, University Medical Center Utrecht, Utrech University, 3584 CG Utrecht, The Netherlands; 6 German Center for Neurodegenerative Diseases (DZNE), Rostock, Germany; 7 Department of Neurology, University of Rostock, Rostock, Germany; 8 German Center for Neurodegenerative Diseases (DZNE), Munich, Germany; 9 Cluster of Systems Neurology (SyNergy), Munich, Germany; 10 Ludwig-Maximilians University Munich, Munich, Germany; 11 Institute for Diabetes and Obesity, Monoclonal Antibody Core Facility and Research Group, Helmholtz Zentrum München, Neuherberg, Germany; 12 Department of Neuropathology, University of Tübingen, Tübingen, Germany

**Keywords:** Frontotemporal dementia, Frontotemporal lobar degeneration, Amyotrophic lateral sclerosis, C9orf72, RAB3, Synaptic vesicles

## Abstract

Hexanucleotide repeat expansion in *C9orf72* is the most common genetic cause of frontotemporal dementia and amyotrophic lateral sclerosis, but the pathogenic mechanism of this mutation remains unresolved. Haploinsufficiency has been proposed as one potential mechanism. However, insights if and how reduced C9orf72 proteins levels might contribute to disease pathogenesis are still limited because C9orf72 expression, localization and functions in the central nervous system (CNS) are uncertain, in part due to the poor specificity of currently available C9orf72 antibodies.

Here, we generated and characterized novel knock-out validated monoclonal rat and mouse antibodies against C9orf72. We found that C9orf72 is a low abundant, cytoplasmic, highly soluble protein with the long 481 amino acid isoform being the predominant, if not exclusively, expressed protein isoform in mouse tissues and human brain. As consequence of the *C9orf72* repeat expansion, C9orf72 protein levels in the cerebellum were reduced to 80% in our series of *C9orf72* mutation carriers (*n* = 17) compared to controls (*n* = 26). However, no associations between cerebellar protein levels and clinical phenotypes were seen. Finally, by utilizing complementary immunohistochemical and biochemical approaches including analysis of human iPSC derived motor neurons, we identified C9orf72, in addition to its association to lysosomes, to be localized to the presynapses and able to interact with all members of the RAB3 protein family, suggestive of a role for C9orf72 in regulating synaptic vesicle functions by potentially acting as guanine nucleotide exchange factor for RAB3 proteins.

In conclusion, our findings provide further evidence for haploinsufficiency as potential mechanism in C9orf72 pathogenesis by demonstrating reduced protein levels in *C9orf72* mutation carriers and important novel insights into the physiological role of C9orf72 in the CNS. Moreover, the described novel monoclonal C9orf72 antibodies will be useful tools to further dissect the cellular and molecular functions of C9orf72.

**Electronic supplementary material:**

The online version of this article (10.1186/s40478-018-0579-0) contains supplementary material, which is available to authorized users.

## Introduction

In 2011, abnormal expansion of a GGGGCC hexanucleotide repeat in a predicted non-coding region of the chromosome 9 open reading frame 72 (*C9orf72)* gene was identified as the most common genetic cause of familial and sporadic forms of frontotemporal dementia (FTD) and amyotrophic lateral sclerosis (ALS) including families in which both conditions co-occur [13, 40]. How the repeat expansion in *C9orf72* contributes to neurodegeneration is currently unresolved. As for other repeat expansion mutations, three non-exclusive pathogenic mechanisms have been proposed: a toxic RNA gain of function by accumulation of transcripts with expanded repeats in RNA foci that bind and sequester specific RNA binding proteins resulting in disruption of their function, a toxic protein gain of function through aberrantly expressed proteins generated by repeat associated non-ATG translation of transcripts with expanded repeats, and haploinsufficiency as consequence of expanded repeats altering expression of their hosting gene.

Based on studies using human tissues and model systems there is evidence for all three mechanisms being in play for *C9orf72* repeat expansions. First, RNA foci composed of mutated sense and antisense C9orf72 transcripts are present in up to 50% of neuronal nuclei in key anatomical regions [12, 13, 15, 32]. RNAs with the extended repeats are reported to fold into G quadruplex structures able to bind several proteins [10, 19, 24, 34]. Second, GGGGCC repeat expansions in sense and antisense transcripts have been shown to act as templates for the synthesis of five aberrantly expressed dipeptide repeat (DPR) proteins by repeat-associated non-ATG translation. Neuronal inclusions composed of these DPR proteins are highly specific neuropathological hallmark features of *C9orf72* mutation carriers [2, 15, 33, 35, 60] and have shown neurotoxic effects in various model systems upon overexpression under artificial AUG start codon [8, 29, 31, 37]. However, so far no consistent correlation of RNA foci and DPR protein pathology with the regional pattern of neurodegeneration and/or presence of TDP-43 pathology, the neuropathological hallmark feature of ALS and FTD including cases with *C9orf72* mutations [36], has emerged despite extensive quantitative analysis [11, 12, 26, 27]. Thus, the pathogenic relevancies of RNA foci and DPR proteins in disease pathogenesis remain to be clarified. Finally, the potential role of haploinsufficiency is supported by the consistent demonstration of decreased *C9orf72* mRNA levels in different tissues including postmortem brain tissue of *C9orf72* mutation carriers [13, 17, 52], motor deficits reported in *C9orf72* knockdown zebrafish [9] and *C.elegans* [50] models as well as contribution of reduced protein levels to neurodegeneration in induced human motor neurons from *C9orf72* mutation carriers [46]. However, the absence of obvious neurologic phenotypes in *C9orf72* knock-out mice argues against a sole role of haploinsufficiency in disease pathogenesis [3, 23, 38, 47], although it remains to be tested whether additional stressors might be required as second hits to induce neurodegeneration in these mouse models.

Further investigations on the potential contribution of C9orf72 haploinsufficiency in disease pathogenesis of ALS and FTD is hampered due to little knowledge about the physiological functions of C9orf72. Based on bioinformatics analysis, C9orf72 is predicted to contain *differentially expressed in normal and neoplastic cells* (DENN) domains [25, 59], that are characteristics of guanine nucleotide exchange factors (GEFs) for specific Rab GTPases (Rabs) [55, 58]. Rabs are key determinants of organelle identities that switch between two conformational states, an inactive form bound to GDP and an active form bound to GTP. The conversion of GDP-bound to GTP-bound Rabs is facilitated by GEFs and results in enrichment of activated Rabs at distinct target membranes where they recruit additional proteins in order to mediate practically all membrane trafficking events in eukaryotes [39]. In agreement with the bioinformatics prediction, recent studies have shown that C9orf72 is part of a protein complex containing Smith-Magenis syndrome chromosomal region candidate gene 8 (SMCR8) and WD repeat containing protein 41 (WDR41) [1, 21, 45, 48, 51, 54, 57], and that this complex possesses GEF activity for RAB8A and RAB39B, two Rabs involved in autophagy [45, 57]. Accordingly, C9orf72 has been found to modulate different steps of the autophagy and endo-lysosomal pathways [1, 21, 45, 48, 51, 54, 57].

Beyond that, insights on functions of C9orf72 and whether C9orf72 repeat expansions result in reduced C9orf72 protein levels are so far limited in part due to poor specificity of currently available C9orf72 antibodies with conflicting and inconsistent results reported on its subcellular distribution and expressed isoforms. Importantly, since the subcellular localization of a predicted GEF protein determines where in the cell specific Rab-GTPases might be activated, it is essential to gain further knowledge on the expression pattern of C9orf72 particularly in the central nervous system (CNS) in order to dissect its functions.

Therefore, the aim of the present study was to generate and characterize novel antibodies against C9orf72 with the goal to investigate the subcellular distribution and function of C9orf72 in the CNS and to test for haploinsufficiency on the protein level in postmortem brain tissue of ALS, ALS/FTD and FTD cases with *C9orf72* repeat expansions.

## Material and methods

### Generation of monoclonal antibodies against C9orf72

Rat monoclonal antibodies (mAbs) were generated against synthesized peptides corresponding to amino acid residues 321–335 (peptide 1), 417–434 (peptide 2), 100–113 (peptide 3), 140–155 (peptide 4) and 184–203 (peptide 5) of human C9orf72 (Protein ID: Q96LT7) coupled to bovine serum albumin (BSA) or ovalbumin (OVA) (Peptide Specialty Laboratories GmbH, Heidelberg, Germany). Lou/c rats were immunized subcutaneously and intraperitoneally with a mixture of 50 μg OVA-coupled peptides, 5 nmol CpG 2006 oligonucleotide (Tib Molbiol, Berlin, Germany), and an equal volume of incomplete Freund’s adjuvant. After a 6 weeks interval, rats were boosted with 50 μg OVA-coupled peptides in PBS. Hyperimmune spleen cells were fused with the mouse myeloma cell line P3X63Ag8.653 using standard procedures. As primary screen supernatants were screened in a solid-phase enzyme-linked immunosorbent assay (ELISA) with the BSA-coupled peptides. Positive supernatants were tested by immunoblot analysis using protein lysates from transiently transfected HEK293T cells. Cells from identified and further characterized mAbs were subcloned at least twice by limiting dilution. Experiments were performed with cell culture supernatants from established clones 12E7 (rat IgG1) against peptide 4, 5F6 (rat IgG1) and 12G10 (rat IgG1) against peptide 5, and 2H7 (rat IgG1) and 15C5 (rat IgG2a) against peptide 2. For generation of mouse monoclonal antibodies, 100 μg of HIS-tagged C9orf72/ SMCR8/ WDR41 complex purified from baculovirus infected insect SF2 cells and 200 μg of poly (I/C) as adjuvant were injected intraperitoneally into 2 months old BALB/c female mice. Five injections were performed at 2 week intervals. Spleen cells were fused with Sp2/0.Agl4 myeloma cells. Hybridoma culture supernatants were tested by ELISA for cross-reaction with GST-tagged C9orf72 purified from BL21(RIL) pRARE *E. coli*. Positive supernatants were then tested by immunofluorescence and immunoblot on lysates from HA-tagged C9orf72 transfected HEK293T cells. Specific cultures were cloned twice on soft agar. One specific clone 1C1 was established (mouse IgG1k), and ascites fluid was prepared by injection of 2 × 10^6^ hybridoma cells into Freund adjuvant-primed BALB/c mice. Epitope mapping revealed that 1C1 recognizes a region between amino acid 170–200 of human C9orf72. All animal experimental procedures were performed according to the European authority guidelines.

### Other antibodies

Other primary antibodies used in this study included: anti-C9orf72 (22637–1-AP, Proteintech Group; 66,140–1-Ig, Proteintech Group; GTX119776, GeneTex; sc-138,763, Santa Cruz; AP12928b, Abgent), anti-synaptoporin (102,002, Synaptic Systems), anti-synaptophysin (101,002, Synaptic Systems; M0776, Dako), anti-GAPDH (MAB374, Millipore), anti-α-tubulin (T5168, Sigma), anti-RAB3 (107,003, Synaptic Systems), anti-RAB3A (HPA003160, Atlas Antibodies), anti-PSD-95 (ab18258, Abcam), anti-LAMP1 (ab24170, Abcam), anti-LAMP2 (10397–1-AP, Proteintech Group), anti-SMCR8 (1D2, homemade), anti-RAB39B (1H1, homemade), anti-Histone H3 (ab21054, Abcam), anti-Cox IV (ab16056, Abcam), anti-myc tag (2276 and 2272, Cell Signaling), anti-FLAG tag (PA1-984B, Pierce), and anti-HA tag (26,183, Pierce or 3F10, Merck).

### Plasmids and constructs

pCMV6 containing C-terminally myc-DDK-tagged cDNAs of human C9orf72 variant 1 (NM_145005), human C9orf72 variant 2 (NM_018325), mouse C9orf72 encoding for isoform 1 (NM_001081343), mouse C9orf72 encoding for isoform 2 (BC026738), C-terminally FLAG-tagged human cDNAs of SMCR8, and Rab proteins were purchased from OriGene. cDNAs for C9orf72 were subcloned into pcDNA5/FRT/TO (Invitrogen) with or without myc-DDK-tag. Optimized cDNA for human N-terminally HA-tagged C9orf72 variant 1 cloned in pcDNA3 was described previously [45] and is available through Addgene.

### Cell cultures and transfection

HEK293T cells were cultured in Dulbecco’s modified Eagle’s medium (DMEM) with Glutamax (Invitrogen) supplemented with 10% (vol/vol) fetal calf serum (FCS, Invitrogen) and penicillin/streptomycin (Invitrogen) at 37 °C with 5% CO_2_. Transfection of cells for immunoblot or immunofluorescence analysis was carried out with Fugene HD (Promega) or calcium phosphate method and indicated plasmids. Medium was exchanged 7 h after transfection and cells were examined 48 h after transfection. For immunoprecipitation experiments 6.25 × 10^5^ HEK293T cells were co-transfected for 24 h with indicated plasmids using Fugene HD (Promega) and further processed as described below.

Motor neurons were generated from human induced pluripotent stem cells (iPSCs) as previously described [28]. Briefly, iPSCs (clone 56c2, Phenocell) were dissociated with Accutase (Invitrogen) and resuspended in differentiation medium N2B27 (DMEM F12, Neurobasal vol:vol supplemented with N2 (Life Technologies), B27 (Life Technologies), Pen-strep 1%, β-mercapthoethanol 0.1% (Life Technologies), ascorbic acid (AA, 0.5 μM, Sigma Aldrich)) with Y-27632 (5 μM, STemGent), SB431542 (40 μM, Tocris), LDN 193189 (0.2 μM, Miltenyi) and Chir-99,021 (3 M, Tocris) for 2 days. Chir-99,021 was maintained from day 0 to day 4 of differentiation and SB431542/LDN 193189 from day 0 to day 7. Differentiation medium was supplemented with retinoic acid (RA, 100 nM, Sigma Aldrich) and smoothened agonist (SGA, 500 nM, Calbiochem) at day 2 of differentiation. At day 7, medium was changed for N2B27 supplemented with RA (100 nM), SAG (500 nM) and BDNF (10 ng/ml). Two days later, DAPT (10 μM, Tocris) was added. Ten days after the beginning of the differentiation, cells were dissociated with Trypsin and plated on poly-ornithin/laminin coated glass coverslips at a density of 33,000 cells per cm^2^ in N2B27 medium containing RA (100 nM), SAG (500 nM), BDNF (10 ng/ml) and DAPT (10 μM). Seven days later, DAPT was withdrawn and replaced by GNDF (10 ng/ml). Cells were analyzed by immunocytochemistry after 30 days of differentiation.

### Immunocytochemistry and immunofluorescence

HEK293T cells: Cells were fixed on coverslips for 15 min in 4% paraformaldehyde (PFA) in PBS, permeabilized for 5 min in 0.25% Triton X-100 in PBS and subsequently incubated for 1 h in blocking buffer (2% fetal bovine serum and 0.1% Triton X-100 in PBS). Coverslips were incubated overnight at 4 °C with the indicated C9orf72 mAbs and anti-myc antibodies diluted in blocking buffer. After washing steps with PBS, coverslips were incubated for 1 h at room temperature with Alexa Fluor 488- or 594-conjugated secondary antibodies (Invitrogen), washed with PBS, incubated in Hoechst 33342 for 5 min to stain nuclei, and mounted onto glass slides using fluorescence mounting medium (Dako Agilent).

Human iPSC derived motor neurons: Glass coverslips with plated cells were fixed in 4% PFA for 10 min and subsequently washed three times with PBS. The coverslips were incubated for 10 min in PBS plus 0.5% Triton X-100 and washed three times with PBS before incubation for 1 h with indicated primary antibodies (1C1, 12E7, SMCR8, LAMP1, LAMP2, RAB39B, RAB3, synaptophysin). Coverslips were washed twice with PBS before incubation with a Cyanine 3-conjugated goat anti-rabbit, anti-mouse or anti-rat and Alexa 488-conjugated donkey anti-rat or anti-mouse secondary antibodies for 1 h, washed with PBS, incubated for 2 min with DAPI (1/10000 dilution in PBS) and rinsed twice with PBS before mounting onto glass slides using ProLong Mountant (Molecular Probes). For quantification, hundred C9orf72-positive vesicles per coverslip were counted and the mean and standard deviation of co-localization was calculated from three independent experiments.

Images were obtained with a fluorescence microscope (Leica) equipped with a CCD camera.

### Immunoprecipitation

Co-transfected HEK293T cells were scraped into radioimmunoprecipitation assay (RIPA) buffer (50 mM Tris–HCI pH 7.6, 150 mM NaCl, 1% NP-40) and centrifuged for 15 min at 18,000 g at 4 °C; 20 μl of pre-washed HA magnetic beads (Dynabeads) was added, and immunoprecipitation was carried out for 1 h at 4 °C with constant rotation. For immunoprecipitation of endogenous C9orf72 from mouse brains, 100 μl of mAb 1C1 was incubated with mouse brain extract in RIPA buffer overnight at 4 °C. Pre-cleared A/G magnetic beads (Life Technologies) were added and immunoprecipitation was carried out for 3 h at 4 °C with constant rotation.

After three washes of beads with washing buffer (50 mM Tris–HCI pH 7.6, 150 mM NaCl, 0.05% Tween), bound proteins were eluted by boiling in SDS–PAGE loading buffer at 95 °C for 5 min and analyzed by immunoblot using the indicated antibodies.

### Mouse tissue

C57BL/6 N mice were bred in our facilities. Animals were euthanized using CO_2,_ organs were removed and further processed for histological and biochemical analysis. Brain tissue from C9orf72 knock-out mice (C9−/−; *n* = 3) described previously [47] and non-transgenic littermates (C9+/+; *n* = 3) were kindly provided by the Pasterkamp lab for knock-out validation experiments of C9orf72 mAbs. All animal experiments were carried out in accordance with the institutional and European authority guidelines.

### Human postmortem tissue

Human post mortem tissues were obtained from the brain banks affiliated with the University of Tübingen and the University of British Columbia. Consent for autopsy was obtained from probands or their legal representative in accordance with local institutional review boards.

The study cohort consisted of 18 cases with a *C9orf72* repeat expansion mutation (C9+) covering the complete clinical spectrum presenting with ALS (*n* = 6), FTD/ALS (*n* = 5) or FTD (*n* = 7) and 33 control cases (C9-) consisting of neurologic disease controls with TDP-43 pathology in the absence of a *C9orf72* repeat expansion mutation clinically presenting with ALS (*n* = 16), ALS/FTD (*n* = 6) and FTD (*n* = 5), three neurologically healthy controls, two Alzheimer’ disease cases and one case with hypoxic encephalopathy. *C9orf72* repeat expansions have been identified by genetic testing using repeat-primed PCR or were inferred from the presence of DPR protein pathology. Details for cases are provided in Additional file 1: Table S1.

### Immunohistochemistry and in situ hybridization

Immunohistochemistry on mouse and human CNS tissue was performed on 2–5 μm thick sections of formalin fixed, paraffin-embedded (FFPE) tissue using the Ventana BenchMark XT automated staining system with the iVIEW DAB detection kit (Ventana).

To establish a protocol for the immunohistochemical detection of C9orf72, mAb 1C1 and 12E7 were first tested on sections from C9+/+ and C9−/− mouse brains (formalin fixation: 24 h) with different dilutions and different antigen pretreatments (boiling for 60 min in CC1 or CC2 buffer (Ventana), or no pretreatment). A specific, knock-out validated signal was obtained in mouse brains for mAb 1C1 using 1:100 dilution and boiling for 60 min with CC1 buffer as pretreatment and this protocol was used for further experiments in this study.

To test for the immunohistochemical detection of C9orf72 in human postmortem FFPE brain tissue, hippocampus, frontal cortex and cerebellum sections (*n* = 3 C9+ and *n* = 3 C9- cases, formalin fixation times ranging from 2 weeks to several months) were stained with the above described protocol for 1C1; however, no immunoreactivity was observed. To test for the influence of formalin fixation times on C9orf72 immunoreactivity, mouse brains (*n* = 2) were fixed in formalin for 12, 24, 48, 72 or 96 h before paraffin embedding and sections stained using the above described 1C1 protocol. Best signals were obtained for 12 and 24 h fixation time points, while a dramatic reduction in immunoreactivity was observed with increasing formalin fixation times. No improvement of 1C1 immunoreactivity signals could be achieved in the > 24 h formalin fixed mouse tissues by testing additional pretreatments (boiling for 90 min in CC1 or CC2 buffer (Ventana), enzymatic digestion with Protease 1 and 2 (Ventana), or formic acid treatment for 5 min).

No specific signal could be detected in mouse FFPE sections using mAb 12E7 with any tested pretreatment in mouse tissue, indicating that its epitope is masked in FFPE tissue. Subsequently, no staining was observed for 12E7 in human postmortem FFPE tissue.

Human C9orf72 specific antibodies 5F6 and 12G10 were tested in parallel on mouse brain sections (used as negative control) and human postmortem hippocampus and cerebellum sections with different dilutions and pretreatments (boiling for 60 min in CC1 or CC2 buffer (Ventana), or no pretreatment). However, all conditions resulted in similar staining profiles in human and mouse tissue due to cross-reactivity with unrelated proteins (Additional file 1: Figure S1). Additional antibodies for immunohistochemistry included polyclonal rabbit anti-synaptoporin, anti-synaptophysin and LAMP1.

In situ hybridization was performed on FFPE mouse sections using the RNAscope® 2.5 HD Reagent Kit-RED (Advanced Cell Diagnostics) according to the user manuals 322,452-USM and 322,360-USM. The target region of the probe (Mm-3110043O21Rik) corresponds to nucleotides 601–1539 of mouse C9orf72 mRNA (NM_001081343).

### RIPA lysates

Mouse tissues and human postmortem brain tissues were homogenized in RIPA buffer (50 mM Tris-HCl, 150 mM NaCl, 1% (*v*/v) octylphenoxy poly(ethyleneoxy)ethanol (IPEGAL), 5 mM EDTA, 0.5% (*w*/*v*) sodium deoxycholate, 0.1% (w/v) sodium dodecyl sulfate (SDS), pH 8.0) at 1 g/2 ml ratio. Lysates were passed through 18 and 21 Gauge needles and sonicated to shear nucleic acids. Cellular debris was removed by centrifugation for 2 min at 3000×g at 4 °C and supernatant collected as RIPA lysate. Transfected cells were scraped into RIPA buffer and subjected to brief sonication on ice. Cell debris was removed by centrifugation for 5 min at 12,000×g at 4 °C. Protein concentration was determined using the Pierce™ BCA Protein Assay Kit (Thermo Fisher Scientific) according to the manufacturer’s recommendations.

### Subcellular fractionations

Cytoplasmic and nuclear proteins from mouse brains were extracted as previously described [18]. Briefly, forebrain tissue from wild-type mice was dounce homogenized in buffer containing 10 mM HEPES, 10 mM NaCl, 1 mM KH_2_PO_4_, 5 mM NaHCO_3_, 5 mM EDTA, 1 mM CaCl_2_, 0.5 mM MgCl_2_ supplemented with protease inhibitors (1 g/10 ml ratio). After 10 min incubation on ice, sucrose was added to a final concentration of 125 mM. An aliquot was collected representing whole lysate, and the remaining homogenate was centrifuged for 10 min at 1000×g at 4 °C. The supernatant was collected as cytoplasmic fraction. The pellet was washed in TSE buffer (10 mM Tris, 300 mM sucrose, 1 mM EDTA, 0.1% IPEGAL supplemented with protease inhibitors) and centrifuged for 12 min at 6250×g at 4 °C. The supernatant was removed and the pellet was washed twice in TSE buffer and centrifuged for 10 min at 4000×g at 4 °C. The final pellet was re-suspended in RIPA buffer containing 2% SDS by sonication and collected as nuclear fraction.

Enrichment of synaptosomal compartments from mouse forebrain was performed according to previously described protocols [7, 16, 20] with minor modifications (see schematic presentation Fig. 2e). Briefly, mouse forebrains were homogenized in 5 volumes of ice-cold buffered sucrose (0.32 M sucrose, 4 mM HEPES-NaOH, pH 7.3) using a Dounce tissue grinder with tight-fitting glass pestle (Wheaton). The homogenate was centrifuged for 5 min at 1000×g at 4 °C. The pellet (P1) containing cellular debris and nuclei was discarded, and the postnuclear supernatant (S1) was centrifuged for 15 min at 12,500×g. The supernatant (S2; synaptosome depleted fraction containing cytosol and microsomes) was removed, and the pellet (P2) representing the crude synaptosomal fraction was washed in buffered sucrose and centrifugation repeated as above. For preparation of crude synaptic vesicles, P2 was resuspended in buffered sucrose, and subjected to osmotic lysis by adding 9 volumes of ice-cold water. After three Dounce strokes, 1 M Tris-HCl pH 7.5 was added to a final concentration of 10 mM. After 30 min incubation on ice, the suspension was centrifuged for 20 min at 25,000×g to yield a lysate pellet (LP1, enriched in presynaptic membrane proteins) and a lysate supernatant (LS1). LS1 was further centrifuged for 2 h at 174000×g to yield LS2 containing the soluble synaptosomal content and LP2 representing the crude synaptic vesicle (SV) fraction.

For preparation of postsynaptic densities (PSDs) the washed P2 fraction was suspended in solution B (0.32 M sucrose, 1 mM NaHCO_3_) by six Dounce strokes. Eight ml of the resuspended P2 was loaded on a discontinuous sucrose gradient (10 ml each of 0.85, 1.0, and 1.2 M sucrose solutions containing 1 mM NaHCO_3_) and centrifuged for 2 h at 82,500×g. The fraction between 1.0 and 1.2 M sucrose containing synaptosomes was collected and diluted with solution B. An equal volume of 1% Triton X-100 in 0.32 M sucrose, 12 mM Tris-HCI pH 8.1 was added, the suspension incubated for 15 min at 4 °C under continuous stirring and then centrifuged for 20 min at 32,800×g. The pellet (P3) was resuspended in solution B, and was layered on a sucrose gradient (composed of 4 ml of 2 M sucrose, 3 ml of 1.5 M sucrose, and 3.0 ml of 1 M sucrose solutions containing 1 M NaHCO_3_) and centrifuged for 2 h at 201,800×g. The fraction between 1.5 and 2.0 M sucrose was collected and diluted to a final volume of 2 ml with solution B. An equal volume of 1% Triton X-100, 150 mM KCI was added and the suspension was centrifuged for 20 min at 201,800×g. The resulting pellet P4 containing PSDs was resuspended in solution B. For immunoblot analysis, 50 μg total protein per fraction were loaded for C9orf72 detection and 20 μg for detection of other marker proteins.

### Biochemical analysis of human postmortem brain tissue

The sequential extraction of proteins with buffers of increasing stringency from fresh-frozen postmortem frontal cortex from *C9orf72* mutation cases (*n* = 2) and controls (*n* = 4) was performed as described previously [36]. Briefly, gray matter was extracted at 5 ml/g (*v*/*w*) with low-salt (LS) buffer (10 mM Tris, pH 7.5, 2 mM EDTA, 1 mM dithiothreitol (DTT), 10% sucrose, and a cocktail of protease inhibitors), high-salt (HS) buffer (50 mM Tris, pH 7.5, 0.5 M NaCl, 2 mM EDTA, 1 mM DTT, 10% sucrose) with 1% Triton X-100, myelin flotation buffer (HS buffer containing 30% sucrose), and Sarkosyl (SARK) buffer (HS buffer + 1% N-lauroyl-sarcosine). The detergent-insoluble material was finally extracted in 0.25 ml/g of urea buffer (7 M urea, 2 M thiourea, 4% 3-[(3-cholamidopropyl)dimethylammonio]-1-propanesulfonate, 30 mM Tris, pH 8.5). Equal amounts of protein fractions per case (10 μl for LS, HS, SARK and UREA) were analyzed by immunoblot. For quantification of C9orf72 levels, RIPA lysates were generated from frozen postmortem cerebellum (*n* = 17 C9+, *n* = 26 C9-) and frontal cortex (*n* = 10 C9-) and analyzed by immunblot analysis as described below. To correlate C9orf72 expression in frontal cortex with levels of neurodegeneration frontal cortex sections were assessed on H&E stains and neurodegeneration/cell death graded as absent (0), mild (1), moderate (2) or severe (3) based on the presence of spongiosis, neuronal loss, and gliosis.

### Immunoblot analysis

Proteins were separated by SDS-polyacrylamide gel electrophoresis (SDS-PAGE). With the exception of immunoblots for the quantification of C9orf72 levels in human lysates, immunoblots were performed using enhanced chemiluminescence detection. Therefore, proteins were transferred to either polyvinylidene difluoride membranes (Millipore) or nitrocellulose membranes (GE Healthcare). Membranes were blocked with Tris buffered saline containing 3–5% non-fat dry milk and incubated with indicated primary antibodies overnight at 4C. Bound antibodies were detected with horseradish peroxidase-conjugated anti-rat IgG (H + L), anti-mouse IgG (H + L), anti-mouse IgG (light chain specific) or anti-rabbit IgG (H + L) and signals were visualized with the chemiluminescence detection reagents Luminata Forte (Millipore) or Amersham ECL Prime (GE Healthcare). Precision Plus Protein Dual Color Standards (Biorad) or PageRule Plus Prestained Protein Ladder, 10 to 250 kDa (ThermoFisher) were used as molecular weight size marker.

Semi-quantitative immunoblot analysis of human tissues was performed using fluorescence detection and the Odyssey® CLx Imaging System (LI-COR Biosciences). Proteins were transferred to nitrocellulose membranes and blocked with Odyssey blocking buffer (LI-COR Biosciences). Antibodies were detected with IRDye800CW or 680RD conjugated anti-rat or anti-mouse IgG (LI-COR). The linear range for C9orf72 detection was determined by serial dilutions of RIPA lysate and consequently 50 μg of RIPA lysates per lane were loaded for analysis. Total protein stains of membranes (BLOT-FastStain, G-Biosciences) were used as loading controls for normalization. This has been shown to be more suitable over measuring housekeeping genes such as GAPDH and is the recommended approach for normalization, particularly when loading of high amount of proteins per lane is necessary as is the case for the detection of C9orf72 [14]. Signal intensities for C9orf72 and total protein stains were analysed using the Image Studio™ software (LI-COR).

### Statistical analysis

Statistical analysis was performed with the GraphPadPrism software (version 7.01 for Windows). Student’s t test (two-tailed) was used for comparison of two groups and one-way ANOVA was used for comparison of multiple groups followed by Tukey honestly significant difference (HSD) post hoc test. Associations between age at death, disease duration, postmortem delay and neurodegeneration with C9orf72 levels were analyzed by Spearman’s rank correlation coefficient. Significance level was set at *p* < 0.05.

## Results

### Characterization of highly specific novel monoclonal C9orf72 antibodies

**Fig. 1:**
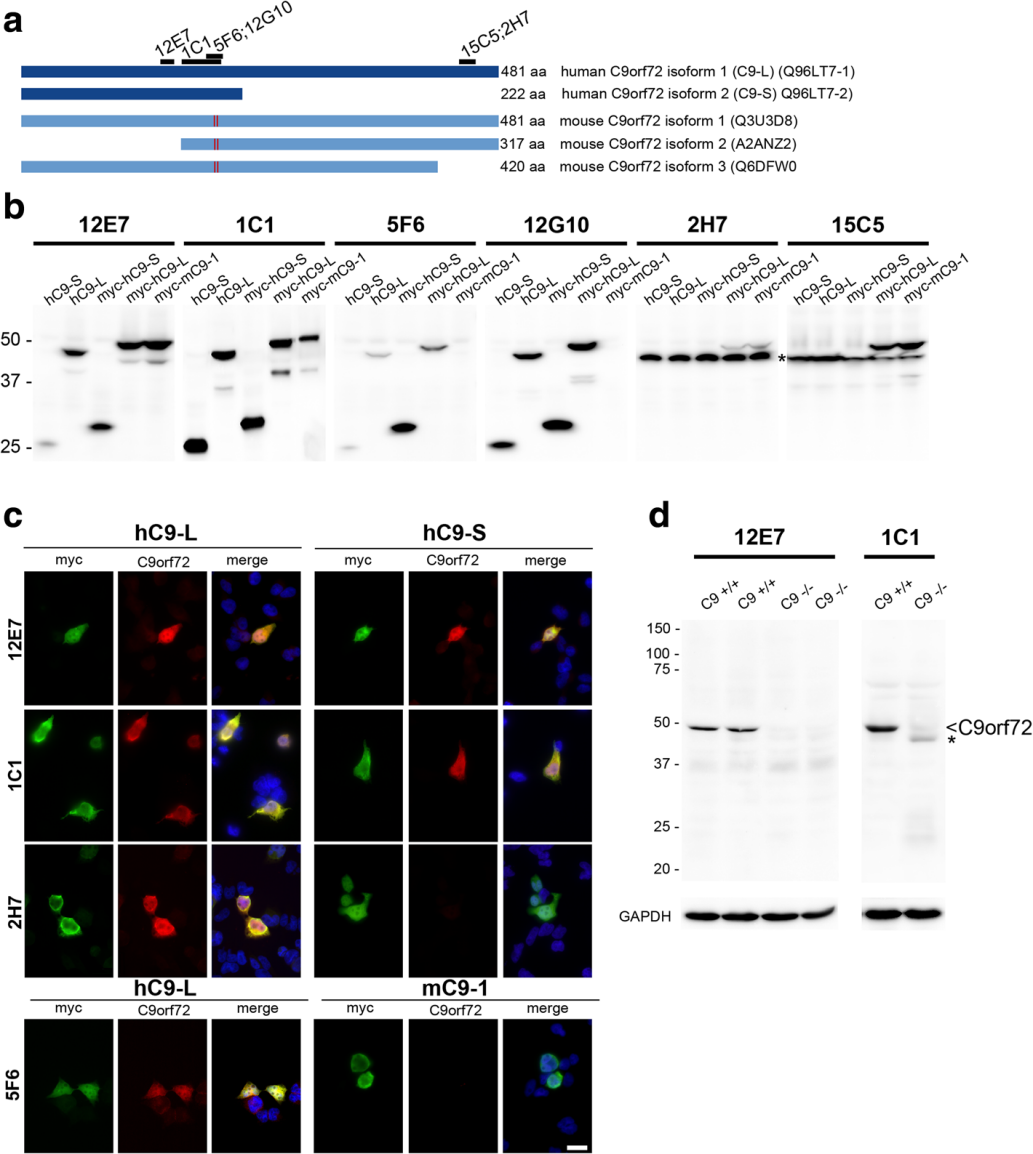
Basic characterization of novel monoclonal antibodies against C9orf72. **a** Schematic representation of postulated human and murine C9orf72 protein isoforms with epitopes recognized by novel monoclonal antibodies (mAbs) against C9orf72. In humans, two C9orf72 protein isoforms are postulated with isoform 1 representing a 481 amino acid protein, also known as long isoform or C9-L (transcribed by transcript variant 2 with the GGGGCC repeat located in the promoter region and transcript variant 3 with the GGGGCC repeat located in the first intron); and isoform 2 representing a 222 amino acid protein, also known as short isoform or C9-S (transcribed by transcript variant 1 with the GGGGCC repeat located in the first intron). In mice, three protein isoforms are postulated, with isoform 1 corresponding in size to human C9-L with 98% similarity on amino acid sequence. The red lines in the murine isoforms illustrate two amino acid changes between the human and mouse C9orf72 sequence in the epitope recognized by mAbs 5F6 and 12G10. **b** Immunoblot analysis of protein lysates of HEK293 cells expressing untagged or myc-DDK-tagged human C9-L and C9-S or myc-DDK-tagged murine C9orf72 isoform 1 (mC9–1) with novel C9orf72 mAbs. Clones 12E7 and 1C1 recognize hC9-S and hC9-L as well as mC9–1. Clones 5F6 and 12G10 specifically recognize human but not mouse C9orf72. Clones 2H7 and 15C5 specifically recognize an epitope in the C-terminus only present in hC9-L and mC9–1 but not hC9-S, however, both mAbs also recognize an unspecific band (asterisk). **c** Double label immunofluorescence for anti-myc (green) and anti-C9orf72 (red) of HEK293 cells transiently expressing myc-DDK-tagged hC9-L, hC9-S or mC9–1 confirms the specificity of the indicated mAbs for specific C9orf72 isoforms or species. Hoechst 33342 staining of nuclei (blue) in the merged images. Scale bar: 20 μm. **d** Immunoblot analysis of total protein lysates from brains of wild-type (C9^+/+^) and C9orf72 knock-out (C9^−/−^) mice. Only a single band around 50 kDa corresponding in size to the murine isoform 1 is detected with mAbs 12E7 and 1C1 in wild-type mice (arrowhead). Note, that this band is completely absent in C9^−/−^ mice, validating the high specificity for C9orf72 of the mAbs 12E7 and 1C1. The weak band labeled with an asterisk seen in C9−/− with the mouse mAb 1C1 represents mouse IgG heavy chain recognized by the anti-mouse IgG (H + L) detection antibody (see Additional file 1: Figure S1b for secondary antibody control). GAPDH is shown as loading control. MW size marker: Precision Plus Protein Dual Color Standards (**b** and **d**)

Novel rat and mouse monoclonal antibodies (mAbs) against C9orf72 were generated and characterized recognizing 4 different epitopes of the human C9orf72 protein sequence (Fig. 1a, Table 1). The specificity of identified mAbs was first demonstrated by immunoblot analysis of protein lysates from HEK293 cells transiently expressing either untagged or myc-DDK-tagged human C9orf72 short (C9-S) and long (C9-L) isoforms as well as murine C9orf72 isoform 1 (mC9–1) and isoform 2 (mC9–1) (Fig. 1b; Additional file 1: Figure S1a). In line with the respective epitopes recognized by the different mAbs, rat clone 12E7 detected C9-S, C9-L and mC9–1; mouse clone 1C1 detected C9-S, C9-L, mC9–1 and mC9–2; rat clones 5F6 and 12G10 specifically labeled human C9-S and C9-L but not murine C9orf72; and rat clones 2H7 and 15C5 labeled C9-L, mC9–1 and mC9–2 but not C9-S. However, clones 2H7 and 15C5 also revealed a strong unspecific band below 50 kDa of the size of untagged C9-L, limiting their usefulness for further studies.

**Table 1:** Summary of basic characterization of novel monoclonal C9orf72 antibodies

	Clone (species)					
	1C1 (mouse)	12E7 (rat)	5F6 (rat)	12G10 (rat)	2H7 (rat)	15C5 (rat)
detection of recombinant proteins						
C9-L	yes	yes	yes	yes	yes	yes
C9-S	yes	yes	yes	yes	no	no
mC9–1	yes	yes	no	no	yes	yes
mC9–2	yes	no	no	no	yes	yes
knock-out validated	yes	yes	na	na	no#	no#

C9-L, long human C9orf72 isoform; C9-S, short human C9orf72 isoform; mC9–1, long murine C9orf72 isoform 1; mC9–2, murine C9orf72 isoform 2; na, not applicable; #unspecific band at similar molecular size of endogenous long C9orf72 isoform

Immunoblot analyses were confirmed by double-label immunofluorescence of HEK293 cells transiently expressing myc-DDK-tagged C9-S, C9-L or mC9–1. Complete co-localization of the diffuse cytoplasmic C9orf72 staining was seen between anti-myc and anti-C9orf72 mAbs 12E7 and 1C1 for C9-S, C9-L, while mAbs 15C5 and 2H7 only recognized C9-L but not C9-S and mAbs 5F6 and 12G10 recognized specifically human but not murine C9orf72 (Fig. 1c, data not shown). Further validation of the specificity of our antibodies to detect C9orf72 was performed by immunoblot analysis of whole brain protein lysates of wild-type mice and C9orf72 knock-out mice. A single band around 50 kDa was obtained for rat mAb 12E7 and mouse mAb 1C1 in wild-type mice corresponding in size to the expected molecular weight of the murine C9orf72 isoform 1 (Fig. 1d; Additional file 1: Figure S1b). Notably, no additional bands were observed at the expected molecular weight size for the postulated murine isoforms 2 and 3, although based on the recognized epitope of 1C1 all murine isoforms should be recognized and absence of isoform 2 was further demonstrated with the C-terminal mAb 15C5 (Additional file 1: Figure S1c). These results indicate that the 481 amino acid long murine isoform 1, which is the equivalent isoform to C9-L in humans, is the predominantly expressed C9orf72 isoform in the mouse CNS. Importantly, this 50 kDa band was absent in lysates of C9orf72 knock-out mice, confirming the specificity of our mAbs 1C1 and 12E7 to detect C9orf72.

Of technical interest, none of the tested commercially available C9orf72 antibodies used in previous studies revealed a comparably high specificity for detecting C9orf72 in our knock-out validation experiments using C9orf72 −/− mouse brain (Additional file 1: Figure S2).

Thus, we generated novel C9orf72 mAbs with knock-out validated specificity as valuable and powerful tools for further analysis of C9orf72 protein expression and localization.

### C9orf72 protein is enriched at the presynapse and co-localizes with a subset of synaptic vesicles in human iPSC-derived neurons in addition to its localization to lysosomes

**Fig. 2:**
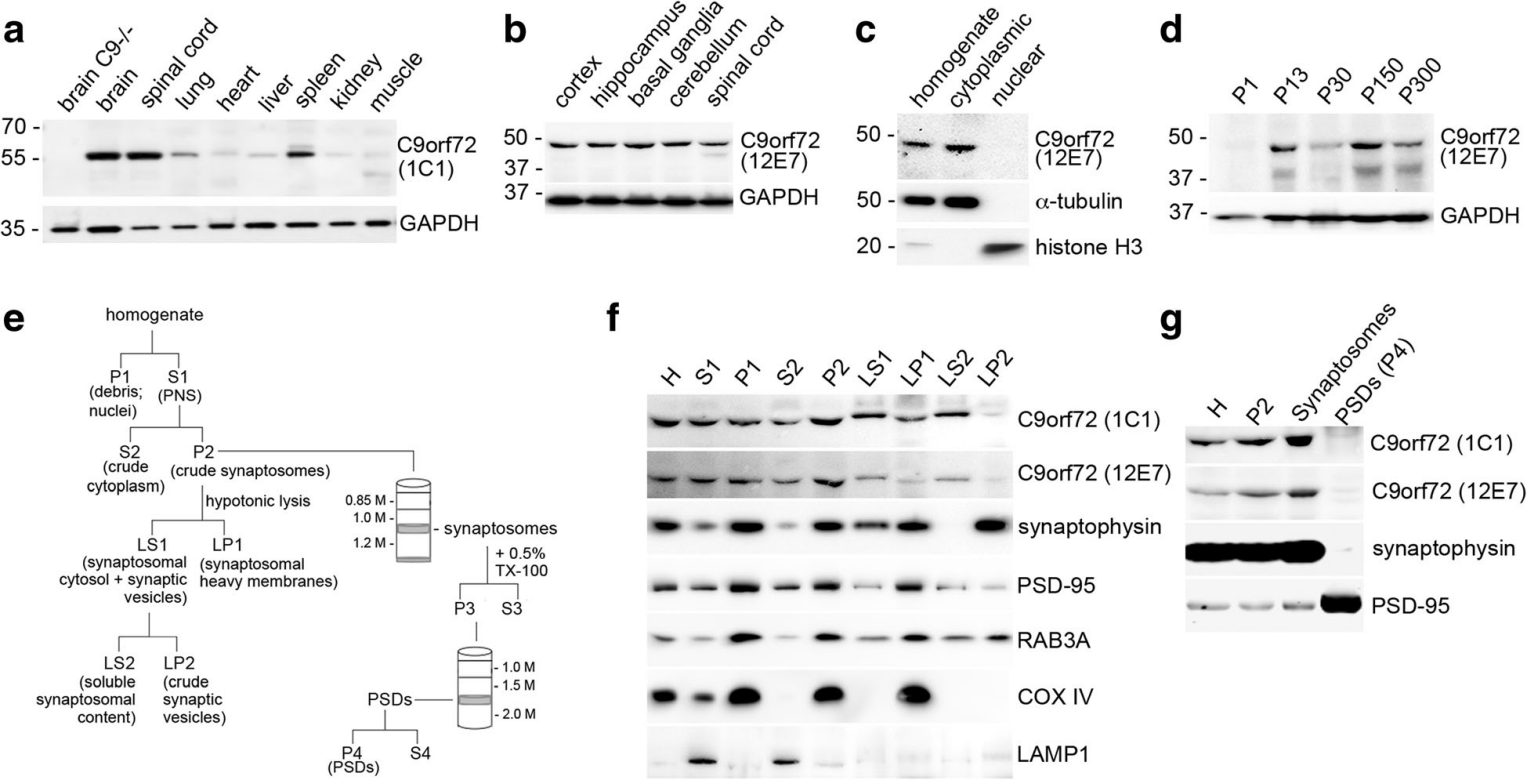
C9orf72 is enriched in synaptosomes. **a** Immunoblot analysis of total protein lysates of different mouse tissues reveals widespread C9orf72 protein expression detected as single band around 50 kDa with highest expression levels in brain followed by spinal cord. GAPDH is shown as loading control. **b** No obvious changes are observed between different brain regions by immunoblot analysis of protein lysates from cortex, hippocampus, striatum, cerebellum and spinal cord. GAPDH is shown as loading control. **c** Immunoblot analysis of nuclear and cytoplasmic protein fractionations extracted from adult mouse brain reveals localization of C9orf72 to the cytoplasm. α-tubulin and Histone H3 are shown to demonstrate purity of the cytoplasmic and nuclear fractions, respectively. **d** Immunoblot analysis and quantification of C9orf72 expression levels over mouse brain development from P1 to P300 showing increase of C9orf72 between P1 and P16. **e** Schematic of the purification protocols for synaptic vesicles and postsynaptic densities (PSDs). Mouse forebrains were homogenized and centrifuged to generate a crude synaptosomal fraction (P2). P2 fractions were fractionated into synaptosomal heavy membranes (LP1), synaptic vesicles (LP2), and synaptic cytosol (LS2) by hypotonic lysis and differential centrifugation. Alternatively, P2 fractions were centrifuged through sucrose gradient to reveal a pure synaptosomal fraction which was further processed to isolate pure PSDs. **f** C9orf72 is detectable in the synaptosomal fraction P2, and is released into the LS2 fraction containing the soluble cytoplasmic content of synaptosomes. **g** C9orf72 is enriched in the pure synaptosomal fraction, but absent from PSD fractions. **f** and **g** The purity of fractions was confirmed with specific marker proteins for synaptic vesicles (synaptophysin), postsynaptic densities (PSD-95), mitochondria (Cox-IV), and lysosomes (Lamp1). MW size marker: PageRule Plus Prestained Protein Ladder (**a**); Precision Plus Protein Dual Color Standards (**b-d, f, g**)

By comparing different tissues of wild-type mice, C9orf72 protein was found to be expressed as long isoform at highest levels in the CNS (brain and spinal cord), at medium levels in tissue of the immune system (spleen) and at low levels in lung, heart, liver, kidney and skeletal muscle (Fig. 2a). These data are in agreement with transcriptome profiles reported in databases [30] and results in transgenic mice with targeted LacZ insertion into the C9orf72 locus [49]. Expression levels of different mouse brain regions did not reveal obvious regional differences (Fig. 2b). In nuclear-cytoplasmic fractionation experiments of mouse brain tissue, C9orf72 was exclusively found in the cytosolic protein fraction (Fig. 2c). Interestingly, when we evaluated the expression levels of C9orf72 in the CNS over a time course from postnatal day 1 to 300 (*n* = 3), we noticed an increase of C9orf72 levels within the first 2 postnatal weeks while after that period no significant changes were observed in C9orf72 expression levels (Fig. 2d). Since this time period with increase of C9orf72 expression coincides with the onset of synaptogenesis and synapse maturation, we speculated that C9orf72 might be localized at the synapse.

To test this hypothesis, subcellular fractionations according to well-established protocols for the enrichment of synaptosomal compartments [7, 16, 20] were performed with adult mouse brain as illustrated schematically in Fig. 2e. Interestingly, a significant fraction of C9orf72 was found to be present in the crude synaptosomal fraction P2 (Fig. 2f and g) and in the pure synaptosomal fraction after sucrose gradient centrifugation (Fig. 2g). After hypotonic lysis of synaptosomal fraction P2 and centrifugation steps, C9orf72 was found to be released into LS2, a fraction enriched for soluble cytoplasmic contents of synaptosomes, while C9orf72 was not present in fraction LP2 enriched for synaptic vesicles (SVs) (Fig. 2f) or in the Triton-extracted PSD fraction P4 (Fig. 2g). Analysis of control proteins for specific fractions revealed expected distribution patterns, with enrichment of synaptophysin in the crude SV fraction LP2 as expected for an integral membrane part of synaptic vesicles, and enrichment of PSD-95 in pure PDS fractions P4 and in LP1 enriched for synaptosomal heavy membranes (Fig. 2f and g), thereby validating the quality of our extractions.

**Fig. 3:**
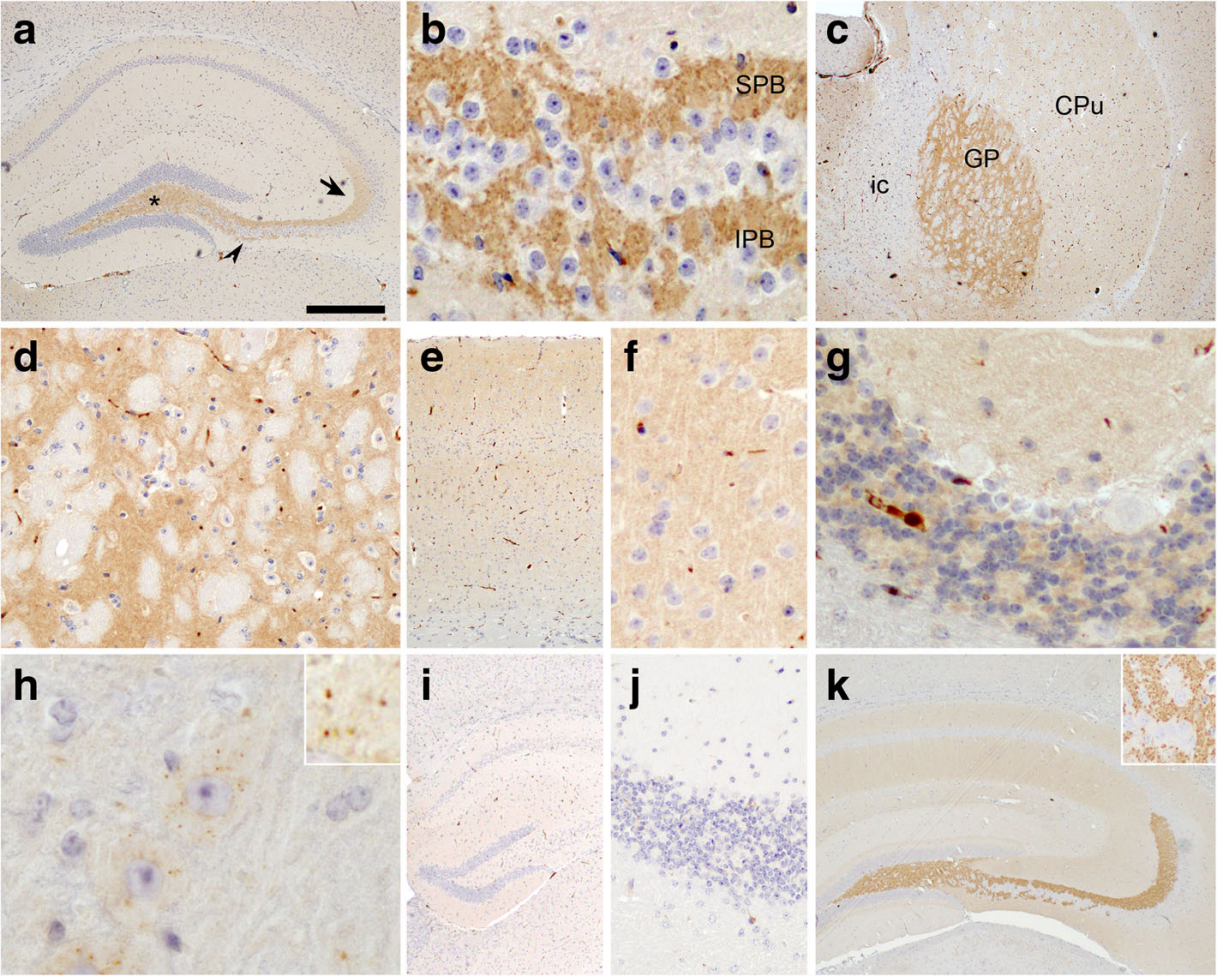
C9orf72 immunohistochemistry reveals synaptic staining pattern with enrichment in hippocampal mossy fiber terminals. Immunohistochemistry with anti-C9orf72 mAb 1C1 (**a-j**); immunohistochemistry with anti-synaptoporin antibody (**k**). In the adult mouse brain (**a-h**), strong immunoreactivity for C9orf72 is seen in the hippocampal mossy fiber system (**a**) with labeling in the hilus (asterisk), stratum lucidum (arrow) and infrapyramidal mossy fiber bundles (arrowhead). (**b**) Higher magnification of punctate staining pattern of mossy fiber terminals in suprapyramidal (SPB) and infrapyramidal (IPB) mossy fiber bundles. Robust staining was also observed in the globus pallidus (GP) (**c** and **d**) while the caudate putamen (CPu) (**c**) and other gray matter regions showed weaker immunoreactivity of the neuropil as shown for frontal cortex (**e** and **f**) and cerebellum with predominant staining in the molecular and granular layer (**g**). No immunoreactivity is seen in the white matter and internal capsule (ic). In addition to punctate neuropil staining, neurons with large cytoplasm such as motor neurons in the spinal cord showed several cytoplasmic puncta (**h**). (**i** and **j**): Specificity of anti-C9orf72 immunohistochemistry was validated by the complete absence of immunoreactivity in brain sections from C9orf72 knock-out mice as shown for hippocampus (**i**) and cerebellum (**j**). Note the strikingly similar staining pattern of the mossy fiber terminals in the hippocampus for C9orf72 (**a**) and for the presynaptic marker protein synaptoporin (**k**). Scale bar: 533 μm (**c**); 400 μm (**a, i, k**); 267 μm (**e**); 80 μm (**d, j,** insert **k**); 40 μm (**b, f, g**); 20 μm (**h**); 6,5 μm (insert **h**)

In line with our biochemical data, immunohistochemistry for C9orf72 performed with mAb 1C1 in mouse brain sections revealed a fine punctate immunoreactivity in the neuropil consistent with a synaptic staining pattern throughout the CNS, although with variable signal intensities (Fig. 3). Specifically, C9orf72 expression was most pronounced in the entire hippocampal mossy fiber system with strong labeling in the hilus, stratum lucidum and the infrapyramidal fiber bundles (Fig. 3a and b). This strikingly resembles the staining pattern seen for other presynaptic marker proteins such as synaptophysin and synaptoporin, a synaptic vesicle protein with an expression pattern mainly restricted to the mossy fiber system (Fig. 3k), a finding further confirmed by double-label immunofluorescence for C9orf72 and synaptoporin (Additional file 1: Figure S3a). A strong neuropil staining was also seen in the globus pallidus (Fig. 3c and d), while weaker immunoreactivity was present in the caudate-putamen (Fig. 3c), throughout the cortex (Fig. 3e and f) and in the molecular layer of the cerebellum (Fig. 3g). In addition to the punctate neuropil staining, particularly large motor neurons in the brain stem and spinal cord (Fig. 3h) demonstrated staining of small dots in the cytoplasm consistent with staining of small vesicles. These vesicles did not stain with antibodies against synaptophysin and LAMP1 (data not shown) and thus their nature remains to be identified. No labeling of nuclei and, no immunoreactivity in the white matter and glial cells was detectable. The expression pattern is in good agreement with our in situ hybridization experiments showing widespread and predominant C9orf72 mRNA expression in neurons with strongest signals in the dentate granule cells but not in glial cells (Additional file 1: Figure S3b). Importantly, the specificity of the observed immunoreactivity for C9orf72 with mAb 1C1 was validated by immunohistochemical analysis of C9orf72 knock-out mouse brain sections showing absence of immunoreactivity (Fig. 3i, j; Additional file 1: Figure S2f). In contrast, all tested commercially available C9orf72 antibodies revealed similar staining intensities and patterns in wild-type and C9orf72 knock-out mice (Additional file 1: Figure S2f).

Unfortunately, we failed to detect reliable immunoreactivity in routinely sampled FFPE human postmortem CNS tissue using the established knock-out validated protocol for mAb 1C1 on mouse tissue. Since we observed that formalin fixation times > 24 h dramatically diminished C9orf72 immunoreactivity signals with 1C1 also in mouse tissue, this is most likely due to the long formalin fixation times (weeks to months) of available human postmortem tissue (for details see Material and Methods). Furthermore, a cross-reactivity of the human specific C9orf72 antibodies 5F6 and 12G10 with additional proteins as shown in brain lysates and tissue sections (Additional file 1: Figure S1d and e) prevented their suitability for immunohistochemical analyses.

**Fig. 4:**
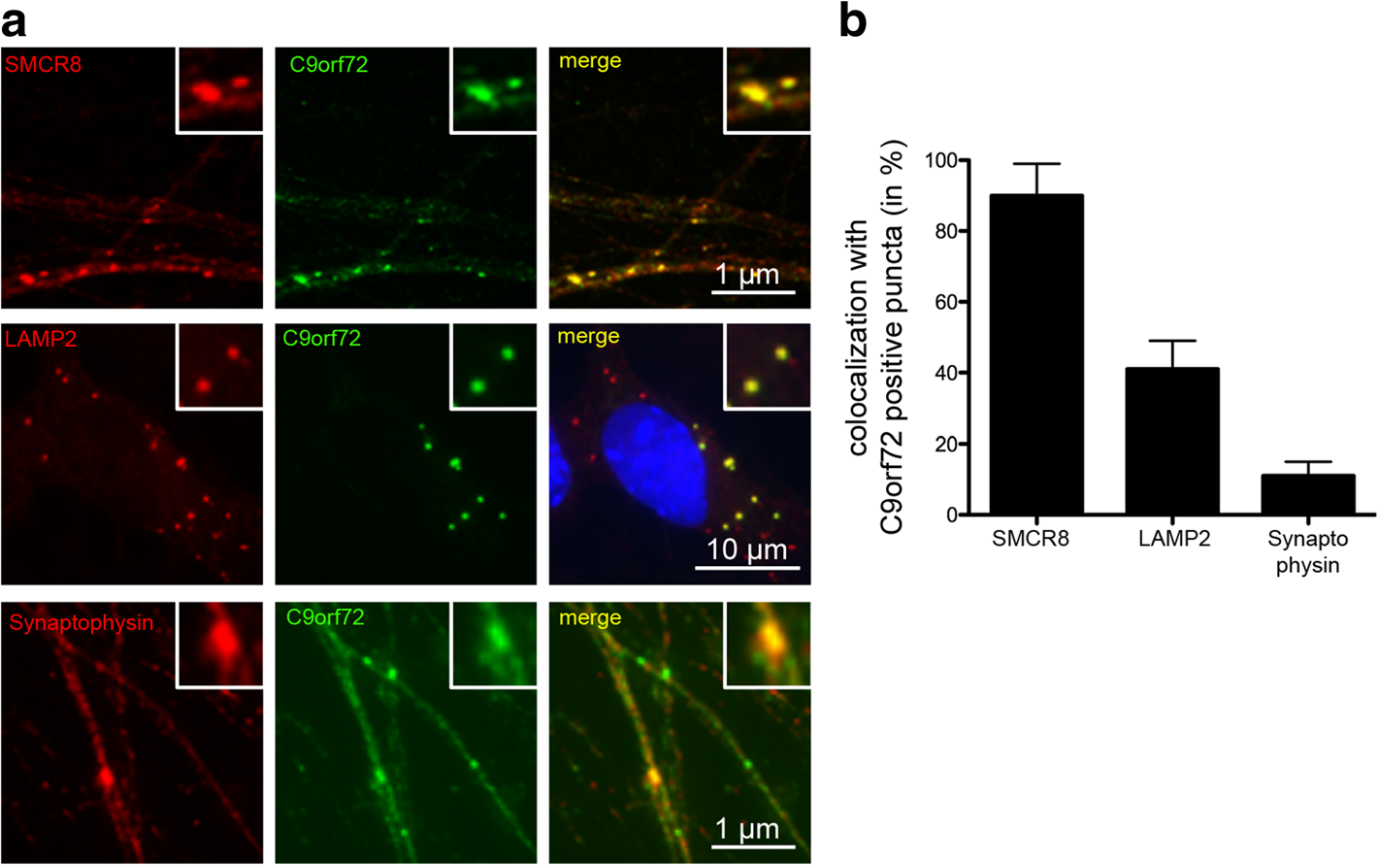
C9orf72 co-localizes with synaptic vesicles in human iPSC derived motor neurons. **a** C9orf72 positive puncta (green) are seen in the axons of 30 day old human iPSC derived motor neurons which consistently co-localize with SMCR8 (red, upper panel) and partially co-localize with LAMP2 as lysosomal marker (red, middle panel) or with the synaptic vesicle marker synaptophysin (red, lower panel). Nuclei are stained with DAPI (blue) in the merged images. C9orf72 labeled with 12E7 antibody in the upper panel and with 1C1 in the middle and lower panel. **b** Graph showing the percentage of C9orf72 positive puncta co-localizing with SMCR8, LAMP2 or synaptophysin. Values are shown as mean ± SD

Thus, to address the localization of C9orf72 in human neurons, we analyzed motor neurons differentiated from human iPSCs. Both knock-out validated C9orf72 antibodies (1C1 and 12E7) revealed presence of C9orf72 in cytoplasmic puncta with complete overlap of signals for both antibodies (Additional file 1: Figure S4a). Double-label immunofluorescence revealed co-localization of C9orf72 with SMCR8 in almost all C9orf72 positive puncta (90 ± 9%) (Fig. 4), consistent with the tight association reported between these two proteins in co-immunoprecipitation experiments [1, 21, 45, 48, 51, 54, 57]. Further analysis showed co-localization of C9orf72 with LAMP1 and LAMP2 (Fig. 4) in a subset of C9orf72 positive puncta (41 ± 8%), indicating that a fraction of C9orf72 is present at lysosomes, consistent with previous observations [1, 46]. Finally, in agreement with our above described findings of a presynaptic localization of C9orf72 in mouse CNS, a fraction (11 ± 4%) of C9orf72 positive puncta co-labelled with synaptophysin used as marker for SVs (Fig. 4) and a comparable co-localization was seen between SMCR8 and synaptophysin (Additional file 1: Figure S4b). However, only a subset (7 ± 3%) of synaptophysin positive SVs was found to co-label with C9orf72, consistent with a transient interaction of C9orf72 with SVs as indicated by our biochemical fractionation experiments. Overall, our data implicate an association of C9orf72 with SVs in mouse and human neurons in addition to its association with lysosomes and vesicles of yet unknown identity.

### C9orf72 interacts and co-localizes with all members of the RAB3 protein family

**Fig. 5:**
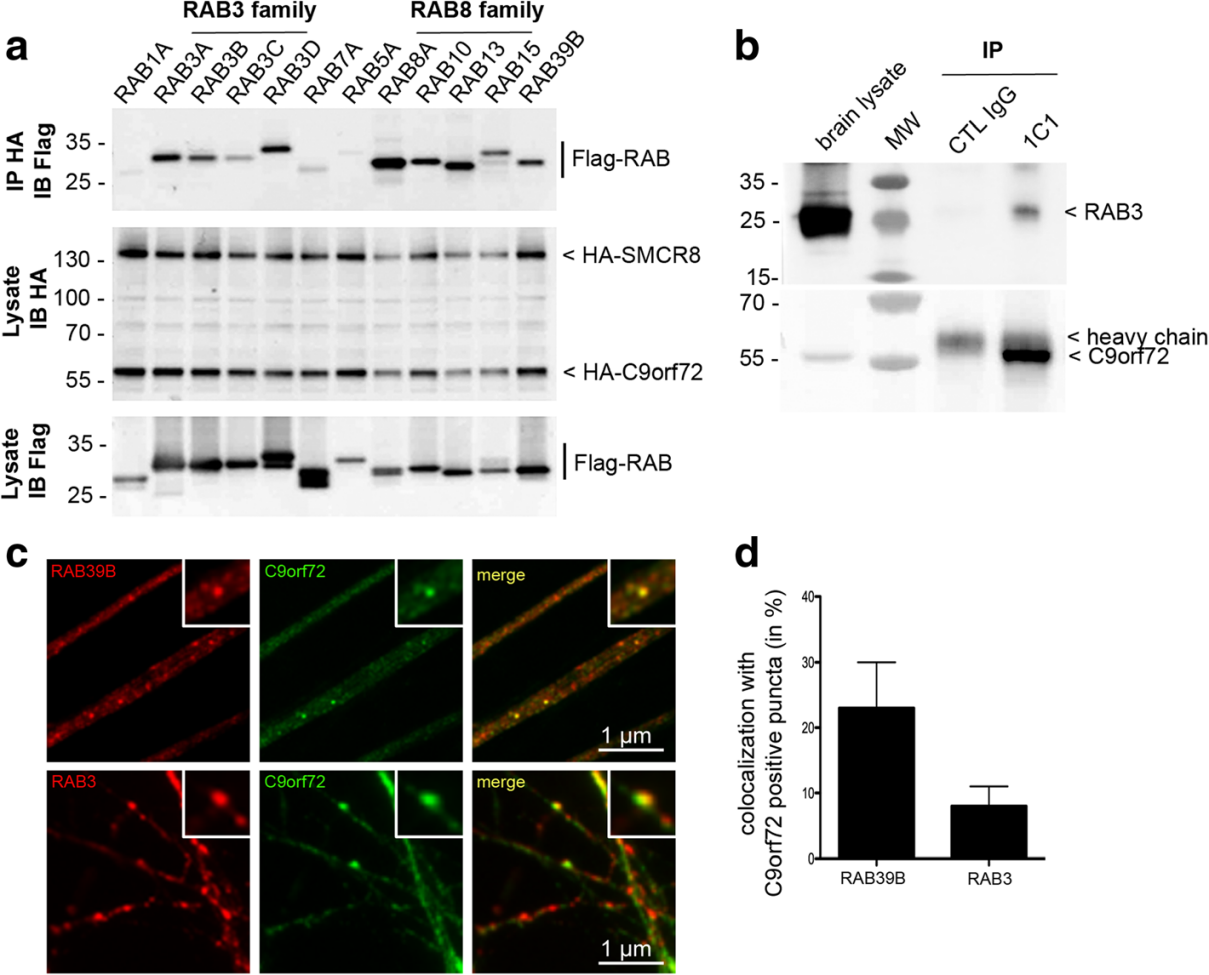
C9orf72 complex interacts with members of the RAB3 protein family. **a** Immunoblot analysis of HA-immunoprecipitated proteins from lysates of HEK293 cells co-expressing HA-tagged human C9orf72 and HA-tagged SMCR8 with various FLAG-tagged Rabs showing co-immunoprecipitation of all members of the RAB3 family with the C9orf72 complex. Other Rabs were used as positive (RAB8 subfamily, RAB39B) or negative (RAB1A, RAB7A, RAB5A) controls based on published reports. **b** Immunoblot against RAB3 of control (IgG alone) or endogenous C9orf72 immunoprecipitated proteins from lysates of adult mouse brain. MW size marker: PageRule Plus Prestained Protein Ladder (**a** and **b)**. **c** Double-label immunofluorescence of 30 day old human iPSC-derived motor neurons showing co-localization of C9orf72 (green) with RAB39B (red, upper panel) or RAB3 (red, lower panel) in a subset of C9orf72-positive puncta. C9orf72 labeled with 12E7 in the upper panel and 1C1 in the lower panel. **d** Graph showing the percentage of C9orf72 positive puncta co-localizing with RAB3 or RAB39B. Values are shown as mean ± SD

Based on our biochemical and immunohistochemical analyses, the subcellular distribution of C9orf72 is consistent with it being a presynaptic terminal associated protein with a transient and reversible interaction with SVs. Given the described function of C9orf72 as GEF for RAB8A and RAB39B, we speculated that C9orf72 might be able to interact with specific Rabs present at SVs. Thus, we performed immunoprecipitation experiments in HEK293 cells co-expressing HA-tagged C9orf72 and SMCR8 and various FLAG-tagged Rabs (Fig. 5a). Excitingly, an interaction of the C9orf72/SMCR8 complex was seen with all members of the RAB3 protein family (RAB3A, RAB3B, RAB3C, RAB3D), which are Rabs abundantly present at SVs (Fig. 2f) and known to play key roles in neurotransmitter release [6]. As controls, interactions of the C9orf72/SMCR8 complex with RAB39B and all members of the RAB8 subfamily (RAB8A, RAB10, RAB13 and RAB15) were identified, comparable to interactions previously described [45]. Importantly, immunoprecipitation of C9orf72 in wild-type mouse brain lysate confirmed the interaction between endogenous C9orf72 and endogenous RAB3 (Fig. 5b). Consistently, double-label immunofluorescence of human iPSC derived motor neurons revealed a partial co-localization of C9orf72 with RAB3 and RAB39B, which was used as positive control (Fig. 5c and d). The interaction of C9orf72 with RAB3 proteins is likely to be transient as only a subset (8 ± 3%) of C9orf72 positive puncta co-localized with RAB3 and conversely, only a subset (5 ± 3%) of RAB3-positive vesicles co-labeled with C9orf72 (Fig. 5d). Thus, our data suggest a potential novel function of C9orf72 by acting as GEF for RAB3 proteins.

### C9orf72 protein expression levels are reduced in the cerebellum as consequence of C9orf72 repeat expansions

To investigate the effect of *C9orf72* repeat expansions on protein expression, we decided to focus for our quantitative immunoblot analysis on cerebellum as brain region, since it is a region known to express high levels of *C9orf72* mRNA [40] and shows consistent and robust changes in transcript levels between *C9orf72* mutation carriers and controls [52, 53]. Moreover and in line with our results showing a predominant neuronal C9orf72 expression, we observed a strong negative correlation (rho = − 0.834, *p* = 0.004, Spearman rank correlation) between C9orf72 protein expression and the level of neurodegeneration/cell death in a pilot experiment on frontal cortex samples from selected cases with no *C9orf72* mutation (Additional file 1: Figure S5), These results highlight the potential bias that neuronal cell loss may blur the interpretation of changes in C9orf72 protein levels. Therefore, we considered that the cerebellum, a region not affected by overt neurodegeneration in ALS and FTD, is best suited for the analysis of *C9orf72* mutation specific consequences on its own protein levels by avoiding misinterpretation of changes related to neuronal cell loss.

**Fig. 6:**
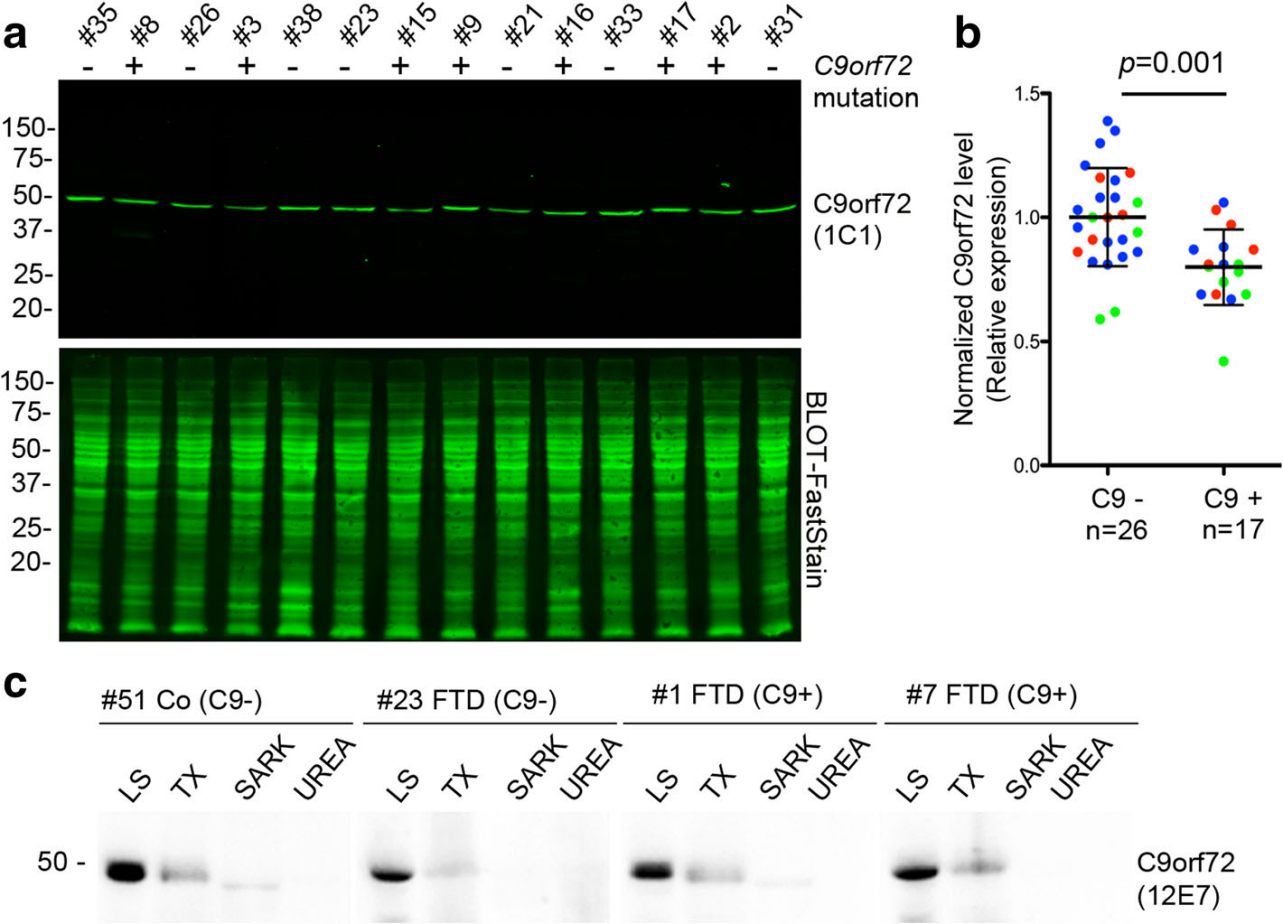
Reduced C9orf72 expression levels in the cerebellum of *C9orf72* mutation carriers. **a** Immunoblot analysis of C9orf72 protein levels in RIPA lysates extracted from frozen cerebellar gray matter of *C9orf72* mutation cases and neurologic controls reveals a single band ~ 50 kDa corresponding in size to the long 481 amino acid isoform of C9orf72 (C9-L). Total protein stains are shown as loading controls. The blot shown is representative of three independent experiments. **b** Quantification of C9orf72 protein levels in the cerebellum of *n* = 17 cases with *C9orf72* repeat expansions (C9+) and *n* = 26 controls (C9-). Dot blot of normalized C9orf72 values with mean and standard deviation shown as line and error bars. Different colors represent clinical phenotypes (green = FTD; red = ALS/FTD; blue = ALS). *p* = 0.001 by Student’s two-tailed, unpaired t test. **c** Proteins were sequentially extracted from frozen frontal cortex of *C9orf72* mutation carriers (C9+) and controls (C9-) with a series of buffers of increasing stringency to receive low salt (LS), high-salt Triton-X-100 (TX), sarkosyl (SARK), and urea protein fractions for immunoblot analysis. Human C9orf72 (C9-L) is present in all cases in the fractions enriched for highly soluble proteins (LS and to lesser extent TX) with no changes observed in solubility between C9+ and C9- cases. MW size marker: PageRule Plus Prestained Protein Ladder (**a** and **c**)

The analyzed cohort consisted of *n* = 17 *C9orf72* mutation carriers covering the complete clinical spectrum from pure ALS, mixed ALS/FTD and pure FTD and *n* = 26 neurologic disease controls (ALS, ALS/FTD and FTD cases without *C9orf72* mutation) with detailed information on each case given in Additional file 1: Table S1. There were no significant differences in the demographics between both cohorts. Immunoblot analysis of total RIPA protein lysates extracted from cerebellum revealed that C9orf72 is a low abundant protein detectable as single band of ~ 50 kDa corresponding in size to C9-L in all samples for mAb 1C1 (Fig. 6a) and 12E7. No band was detectable corresponding to the molecular size of the predicted human C9-S isoform, although both antibodies are able to detect C9-L and C9-S isoforms expressed in HEK293 cells (Fig. 1) with comparable sensitivity, implying that the 481 amino acid isoform (C9-L) is the main and predominant protein isoform expressed in the human CNS as in the mouse CNS. Importantly, subsequent quantitative analysis of C9-L levels normalized to total protein stains revealed a ~ 20% reduction of C9-L levels in cases with *C9orf72* repeat expansions compared to controls (*p* = 0.001) (Fig. 6b). There were no significant differences in C9orf72 protein levels within each cohort between cases presenting clinically with either ALS, FTD/ALS or FTD and there were no associations between cerebellar C9orf72 protein levels and disease duration, age at death or post-mortem delay.

Finally, to further analyze for potential biochemical alterations of C9orf72 in mutation carriers, proteins were sequentially extracted from frozen frontal cortex from cases with or without a *C9orf72* repeat expansion, using a series of buffers with an increasing ability to solubilize proteins. C9-L was found to be present in the protein fractions enriched for soluble proteins (low salt and Triton X-soluble fractions) but not in the fractions enriched for insoluble proteins (sarkosyl and urea soluble fractions). There were no changes in solubility between *C9orf72* mutation carriers and controls (Fig. 6c). Overall, these results demonstrate that C9orf72 is a low abundant, cytoplasmic soluble protein in the human CNS with C9-L being expressed as the predominant protein isoform and with reduced protein levels as consequence of *C9orf72* repeats expansions.

## Discussion

An abnormal hexanucleotide expansion in a non-coding region of *C9orf72* is the most common genetic cause of ALS and FTD [13, 40]. The mechanisms of how this mutation contributes to neurodegeneration are unclear with haploinsufficiency of C9orf72 functions suggested as one potential disease mechanism based on consistently observed reduced RNA transcript levels in tissues of *C9orf72* mutation carriers [13, 17, 52]. However, due to little knowledge on C9orf72 expression, localization and functions particularly in the CNS, further insights if and how reduced C9orf72 proteins levels might contribute to disease pathogenesis are currently limited.

Here, we generated and characterized novel knock-out validated C9orf72 monoclonal rat and mouse antibodies that indicate that C9orf72 is predominantly, if not exclusively, expressed as the long 481 amino acid isoform in human and mouse tissues. Utilizing these antibodies we detected reduced C9orf72 protein levels in the cerebellum as consequence of *C9orf72* repeat expansions in our studied cohort of *C9orf72* mutation carriers. Finally, we identified C9orf72 to be localized to the presynapses and able to interact with members of the RAB3 protein family, suggestive of a role for C9orf72 in regulating SV functions by potentially acting as GEF for RAB3. These findings provide important novel insights into the physiological role of C9orf72 in the CNS, with significant implications for future studies addressing the potential contribution of haploinsufficiency in C9orf72 disease pathogenesis as well as therapeutic strategies.

Based on C9orf72 RNA transcripts two human and three murine C9orf72 protein isoforms have been predicted. Previous biochemical studies of endogenous C9orf72 protein expression have provided inconsistent results with reported presence of only C9-L [41, 53] or C9-L and C9-S [56] as well as presence of only murine variant 1 [23, 38] or all three mouse variants [4]. This observed variability is likely due to use of antibodies that lack sufficient specificity as illustrated by our results of commercially available C9orf72 antibodies on C9orf72 knock-out mouse brain tissue (Additional file 1: Figure S2), a technical limitation also reported by others [42, 53]. Here, utilizing knock-out validated C9orf72 mAbs generated against epitopes that allow detection of all predicted isoforms in vitro, one band was observed in examined mouse and human tissues corresponding in size to human C9-L and the corresponding variant 1 of murine C9orf72. While this does not exclude that additional C9orf72 protein isoforms might be present at amounts below detection limit of our immunoblot assay, our data indicate that the long 481 amino acid isoform is by far the most predominantly expressed C9orf72 protein isoform in mouse and human. Biochemical analyses of C9-L extracted from human postmortem tissue revealed no changes in solubility of C9-L between C9orf72 mutation carriers and controls, which is consistent with a previous report [56]. However, we found that C9-L is mostly a soluble protein with presence in LS and TX fractions, in contrast to a previous study reporting significant amount of C9orf72 in TX-insoluble, urea-soluble protein fractions [56]. Discrepancies between both studies might be explained by different antibody specificities and differences in extraction methods.

Reduced C9-L levels in postmortem brain tissue of *C9orf72* mutation carriers have been reported for some cortical regions but surprisingly not for cerebellum [41, 53, 56], the region with highest *C9orf72* RNA expression [40] and consistently reported reduced transcript levels in *C9orf72* mutation carriers [17, 52, 53]. However, the overall interpretation of these data in providing evidence for haploinsufficiency on the protein level has been complicated by non-specific binding of antibodies, insufficient statistical power due to small sample size and the risk that reduced protein levels observed in cortical regions might be influenced by neurodegenerative changes instead of *C9orf72* mutation specific consequences. This latter limitation is underpinned by our finding of a strong negative correlation between presence of neurodegeneration/cell death and C9orf72 levels in cortical regions. Therefore, we focused for our quantitative immunoblot analysis on the cerebellum, a region without overt neurodegeneration in ALS and FTD. Utilizing our novel knock-out validated mAbs we found cerebellar C9-L protein levels reduced to ~ 80% in our cohort of *C9orf72* mutation carriers (*n* = 17) compared to controls (*n* = 26). Notably, the observed degree of protein reduction is in good agreement with the reported decrease to 70% for RNA transcripts encoding for the long isoform in C9orf72 mutation carriers [13, 52]. No associations between clinical phenotypes (ALS, ALS/FTD or FTD), age at onset and disease duration with cerebellar *C9orf72* protein levels were seen. This is consistent with the reported lack of associations between cerebellar transcript levels and clinical features [52]; however, the number of cases per clinical subgroup in our cohort might have been too small to detect subtle associations with protein levels and this should be further addressed in larger cohorts.

While our data expand the evidence for reduced protein expression as a consequence of *C9orf72* hexanucleotide expansions, it remains to be established if and how reduced proteins levels might contribute to disease pathogenesis. The fact that reduced protein levels are described in unaffected and affected brain regions in *C9orf72* mutation carriers, implies that reduced C9orf72 levels are not causing neurodegeneration per se, an interpretation further supported by the absence of obvious neurological phenotypes in C9orf72 knock-out mice [3, 23, 38, 47]. However, these data would be in line with a scenario that different cells and/or neuronal subpopulations might have distinct vulnerabilities in tolerating reduced C9orf72 levels which might trigger neurodegeneration in combination with additional stressors [45], and/or as consequence of a cooperative gain- and loss-of-function mechanism of repeat expansions as recently proposed [46].

One crucial prerequisite to further address the potential role of reduced C9orf72 levels in disease pathogenesis is to gain further insights on its physiological function in the CNS. Several recent studies have demonstrated that C9orf72 interacts with the SMCR8 protein and regulates the endo-lysosomal and autophagy pathways [1, 21, 45, 48, 51, 54, 57]. Our immunofluorescence data of human iPSC derived human motor neurons are consistent with these studies by demonstrating endogenous co-localization of almost all C9orf72-immunoreactive vesicles with SMCR8 and co-localization of ~ 40% of C9orf72-positive vesicles with the lysosomal marker LAMP2. Most excitingly, our complementary histological and biochemical approaches provided strong evidence for an additional role of C9orf72 at presynaptic terminals by acting as putative GEF for members of the RAB3 protein family*.* Immunohistochemically, we found a predominantly presynaptic staining pattern of C9orf72 in mouse brains most prominently in the synapse-rich hippocampal mossy fiber system, where it co-localized with SV marker proteins. No immunoreactivity was observed in glial cells in mouse tissue in agreement with our in situ hybridization data and with the predominant neuronal expression of C9orf72 reported in transgenic mice with targeted LacZ insertion into the *C9orf72* locus [49] under physiological conditions. However, in future studies it will be interesting to investigate whether cellular expression patterns might change under neuroinflammatory and neurodegenerative conditions. In accordance with our immunohistochemical findings, C9orf72 was present in biochemical preparations of synaptosomes and particularly in fractions enriched for soluble cytosolic contents of synaptic vesicles, which is suggestive for a protein able to reversibly and transiently interact with SVs [22]. This interpretation is further supported by the co-localization of C9orf72 in a subset of synaptophysin-positive vesicles in human iPSC derived motor neurons. Like all other trafficking steps in eukaryotes, SV cycle and presynaptic neurotransmitter release is governed by specific Rabs [6] with the most abundant Rabs in neurons represented by homologues of the RAB3 protein family specifically localizing to SVs and with well studied roles in regulating/modulating neurotransmitter release [43, 44]. Our findings of an interaction of C9orf72 with RAB3 family members by co-immunoprecipitation experiments and double-label immunofluorescence indicate that C9orf72 might act as GEF for RAB3 thereby modulating the SV cycle. In support of this interpretation, it is noteworthy that subtle cognitive and imaging alterations observed in a recent study of presymptomatic *C9orf72* mutation carriers were proposed to represent an early and non-evolving phenotype related to neurodevelopmental effects of *C9orf72* mutation [5]. However, the exact role of C9orf72 as potential GEF in modulating neurotransmission and other steps of the SV cycle which also includes Rabs involved in endosomal and autophagosomal functions [6] will require further functional investigation.

One limitation of our study is that the subcellular distribution of C9orf72 in postmortem human brain tissues could not be investigated immunohistochemically due to the lack of immunoreactivity in human FFPE tissue using the knock-out validated protocol successfully established in mouse FFPE tissue. This might be potentially explained by protein degradation due to postmortem delay. However, we observed no association between C9orf72 levels and postmortem delay and mouse tissue with different PM delay mimics in our biochemical analysis. An additional and perhaps more likely explanation seems to be related to formalin fixation times. For mouse tissue we observed decreasing immunoreactivity signals for formalin fixation times > 24 h, while the available human postmortem tissue was routinely fixed for several weeks up to month. This issue needs to be addressed in future studies using differently processed autopsy and perhaps biopsy tissues if available.

## Conclusions

In summary, our data provide evidence for haploinsufficiency at the protein level in *C9orf72* mutation carriers and novel insights into the physiological role of C9orf72 at the presynapse with a potential role as GEF for RAB3 involved in SV exocytosis. These findings have significant implications for future studies aimed at addressing C9orf72 pathogenesis as well as therapeutic strategies. Furthermore, these novel mAbs against C9orf72 will be useful tools to further dissect the cellular and molecular functions of C9orf72.

## Additional file

Additional file 1: **Table S1.** Demographic, clinical and pathological diagnosis of cases used in this study; **Figure S1.** Further characterization of novel monoclonal C9orf72 antibodies; **Figure S2.** Commercially available C9orf72 antibodies tested on C9orf72 knock-out brain tissue; **Figure S3.** C9orf72 double-label immunofluorescence and C9orf72 in situ hybridization; **Figure S4.** Immunofluorescence of human iPSC derived motor neurons; **Figure S5.** Immunoblot analysis of C9orf72 expression levels in frontal cortex. (PDF 14467 kb)
