# Supplementary material for: Novel antibodies reveal presynaptic localization of C9orf72 protein and reduced protein levels in C9orf72 mutation carriers
Source: Acta Neuropathol Commun. 2018 Aug 3;6:72. doi: 10.1186/s40478-018-0579-0 (PMC6091050; doi:10.1186/s40478-018-0579-0)
Supplement: Supplementary file 1 — Table S1. Demographic, clinical and pathological diagnosis of cases used in this study; Figure S1. Further characterization of novel monoclonal C9orf72 antibodies; Figure S2. Commercially available C9orf72 antibodies tested on C9orf72 knock-out brain tissue; Figure S3. C9orf72 double-label immunofluorescence and C9orf72 in situ hybridization; Figure S4. Immunofluorescence of human iPSC derived motor neurons; Figure S5. Immunoblot analysis of C9orf72 expression levels in frontal cortex. (PDF 14467 kb) [file 40478_2018_579_MOESM1_ESM.pdf]

Novel antibodies reveal presynaptic localization of C9orf72 protein and reduced protein levels in *C9orf72* mutation carriers

Petra Frick[1], Chantal Sellier[2], Ian R. A. Mackenzie[3], Chieh-Yu Cheng[1], Julie Tahraoui-Bories[4], Cecile Martinat[4], R. Jeroen Pasterkamp[5], Johannes Prudlo[6,7], Dieter Edbauer[8,9,10], Mustapha Oulad-Abdelghani[2], Regina Feederle[8,9,11], Nicolas Charlet-Berguerand[2], Manuela Neumann[1,12]

Supplementary online material:

Supplementary table 1
Supplementary figure 1-5

Table S1: Demographic, clinical and pathological diagnosis of cases used in this study

case no	C9orf72 mutation	Clinical diagnosis/ phenotype	NP diagnosis	Sex	Age at death	Disease duration	PM delay	Used for immunoblot quantification	Used for sequential extraction
1	yes	FTD	FTLD-TDP	F	66	7	na	Ce	FC
2	yes	FTD	FTLD-TDP	F	52	4	48	Ce	
3	yes	FTD	FTLD-TDP	F	51	2	8-38	Ce	
4	yes	FTD	FTLD-TDP, AD, CVD	M	74	18	56-86	Ce	
5	yes	FTD	FTLD-TDP	M	61	10	32-62	Ce	
6	yes	FTD	FTLD-TDP	F	53	12	8-38	Ce	
7	yes	FTD	FTLD-TDP	M	41	6	na	/	FC
8	yes	FTD/MND	FTLD/ALS-TDP	F	61	3	120	Ce	
9	yes	FTD/MND	FTLD/ALS-TDP	M	66	1	22	Ce	
10	yes	FTD/MND	FTLD/ALS-TDP	M	61	4	72-96	Ce	
11	yes	FTD/MND	FTLD/ALS-TDP	M	55	3	32-62	Ce	
12	yes	FTD/MND	FTLD/ALS-TDP	F	69	8	56-86	Ce	
13	yes	MND	ALS-TDP	M	66	7	48	Ce	
14	yes	MND	ALS-TDP	M	60	3	40	Ce	
15	yes	MND	ALS-TDP	F	59	2	<14	Ce	
16	yes	MND	ALS-TDP	F	70	2	32-62	Ce	
17	yes	MND	ALS-TDP	F	51	1	8-38	Ce	
18	yes	MND	ALS-TDP	F	59	1	56-86	Ce	
19	no	FTD	FTLD-TDP	F	63	5	17	Ce	
20	no	FTD	FTLD-TDP	F	59	9	39	Ce	
21	no	FTD	FTLD-TDP	M	65	1	na	Ce	
22	no	FTD	FTLD-TDP	M	69	6	21	Ce, FC	FC
23	no	FTD	FTLD-TDP	M	64	5	48	Ce, FC	FC
24	no	FTD/MND	FTLD/ALS-TDP	M	84	1	na	Ce	
25	no	FTD/MND	FTLD/ALS-TDP	F	74	3	na	Ce	
26	no	FTD/MND	FTLD/ALS-TDP	M	60	2	na	Ce	
27	no	FTD/MND	FTLD/ALS-TDP	F	74	2	11	Ce	
28	no	FTD/MND	FTLD/ALS-TDP	M	45	3	8	Ce	
29	no	FTD/MND	FTLD/ALS-TDP	M	75	2	8	Ce	
30	no	MND	ALS-TDP	F	66	4	54	Ce	
31	no	MND	ALS-TDP	M	60	13	na	Ce	
32	no	MND	ALS-TDP	F	52	2	16	Ce	
33	no	MND	ALS-TDP	F	70	1	14	Ce, FC	
34	no	MND	ALS-TDP	M	67	na	48	Ce	
35	no	MND	ALS-TDP	F	72	3	na	Ce	
36	no	MND	ALS-TDP	M	47	2	14	Ce	
37	no	MND	ALS-TDP	F	67	5	73	Ce	
38	no	MND	ALS-TDP	M	50	4	17	Ce	
39	no	MND	ALS-TDP	M	76	2	3	Ce	
40	no	MND	ALS-TDP	M	63	1	16	Ce	
41	no	MND	ALS-TDP	F	64	5	na	Ce	
42	no	MND	ALS-TDP	F	65	3	na	Ce	
43	no	MND	ALS-TDP	M	61	2	96	Ce	
44	no	MND	ALS-TDP	M	82	5	19	Ce	

45	no	MND	ALS-TDP	M	76	1	32	FC	
46	no	dementia	AD	F	78	10	21	FC	
47	no	dementia	AD	F	82	na	37	FC	
48	no	/	Hypoxic encephalopathy	M	75	/	27	FC	
49	no	/	Control	M	60	/	7	FC	FC
50	no	/	Control	M	63	/	33	FC	
51	no	/	Control	M	53	/	22	FC	FC

AD, Alzheimer disease; Ce, cerebellum; CVD, cerebrovascular disease; F, female; FC, frontal cortex; FTD, frontotemporal dementia; FTLD-TDP, frontotemporal lobar degeneration with TDP-43 pathology; ALS-TDP, amyotrophic lateral sclerosis with TDP-43 pathology; M, male; MND, motor neuron disease; PM, post mortem; na, not available;

Figure S1

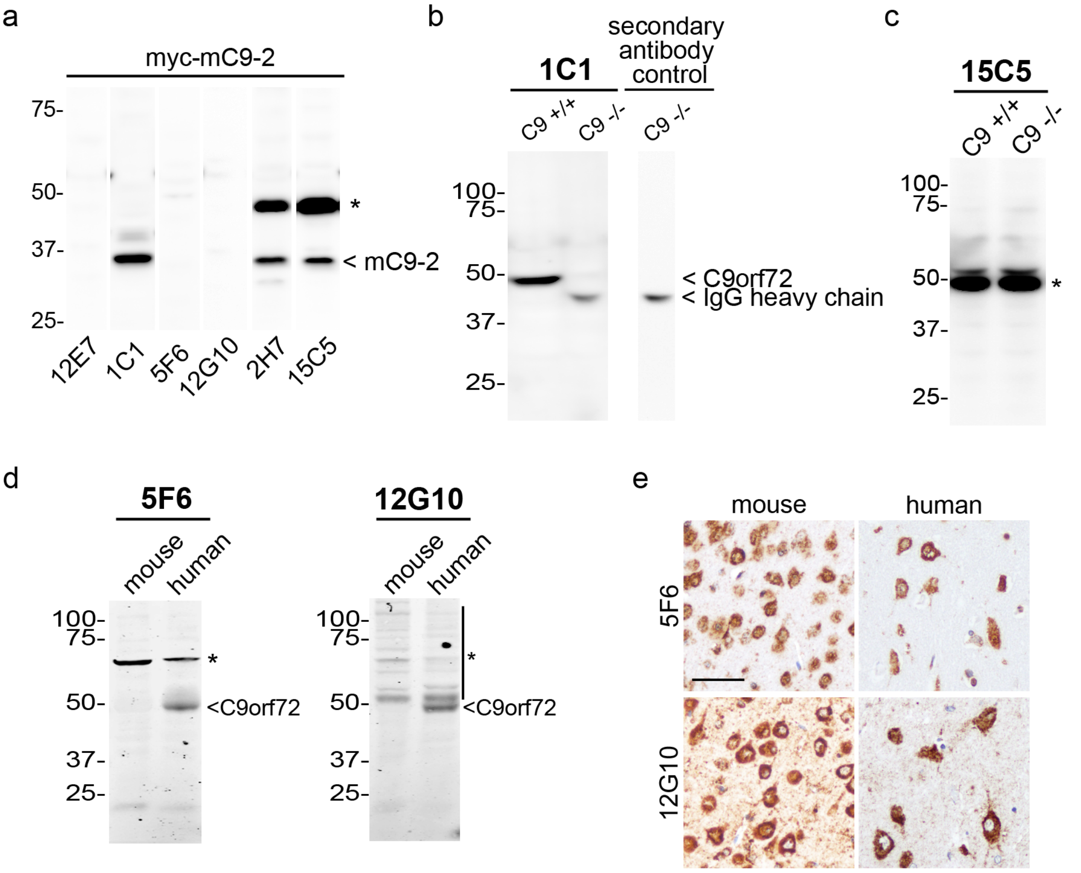

Further characterization of novel monoclonal C9orf72 antibodies

(**a**) Immunoblot analysis of protein lysates of HEK293 cells expressing myc-DDK-tagged murine C9orf72 isoform 2 (mC9-2) with novel C9orf72 mAbs. As expected based on the epitopes recognized by the antibodies, clones 1C1, 12H7 and 15C5 recognize recombinant mC9-2. Asterisk labels an unspecific band ~50 kDa recognized by clones 2H7 and 15C5. (**b**) Immunoblot analysis of total protein lysates from brains of wild-type ($C9^{+/+}$) and C9orf72 knock-out ($C9^{-/-}$) mouse with mouse mAb 1C1 and corresponding secondary antibody only control. The band around ~50 kDa in C9+/+ mice corresponding in size to the murine isoform 1 is completely absent in C9-/- mice. Note that the fuzzy band seen in the C9-/- lysate is also present when only the secondary antibody (HRP-coupled goat anti-mouse IgG (H+L)) was applied, thereby demonstrating that this unspecific band corresponds to endogenous IgG heavy chain. (**c**) Immunoblot analysis of total protein lysates from brains of wild-type ($C9^{+/+}$) and C9orf72 knock-out ($C9^{-/-}$) mouse with mAb 15C5. As in HEK293 lysates, an unspecific band ~50 kDa is detected in mouse tissue lysates (asterisk) which overlays in size with murine C9orf72 isoform 1, therefore limiting its usefulness for further analysis. However, note that no additional band corresponding to mouse isoform 2 is detected with this C-terminal antibody. (**d**) To further characterize the human C9orf72 specific mAbs 5F6 and 15C5 total protein lysates from brains of wild-type mouse and from frontal cortex of human postmortem tissue (case # 51) were analysed by immunoblot. As expected, both antibodies specifically detect human C9orf72 (arrowhead) but not murine C9orf72 in brain lysates. However, unspecific cross reactivity with labelling of other proteins (*) is seen with both antibodies. (**e**) As a result of non-specific binding of the antibodies to unrelated proteins similar immunoreactivity is seen in immunohistochemical stainings of formalin fixed paraffin embedded sections from mouse brain (used as negative control) and human brain tissue which prevented the antibodies from being suitable for further immunohistochemical analysis of human tissue. Scale bar in **e**: 50 µm. MW size marker: Precision Plus Protein Dual Color Standards (**a-d**).

Figure S2

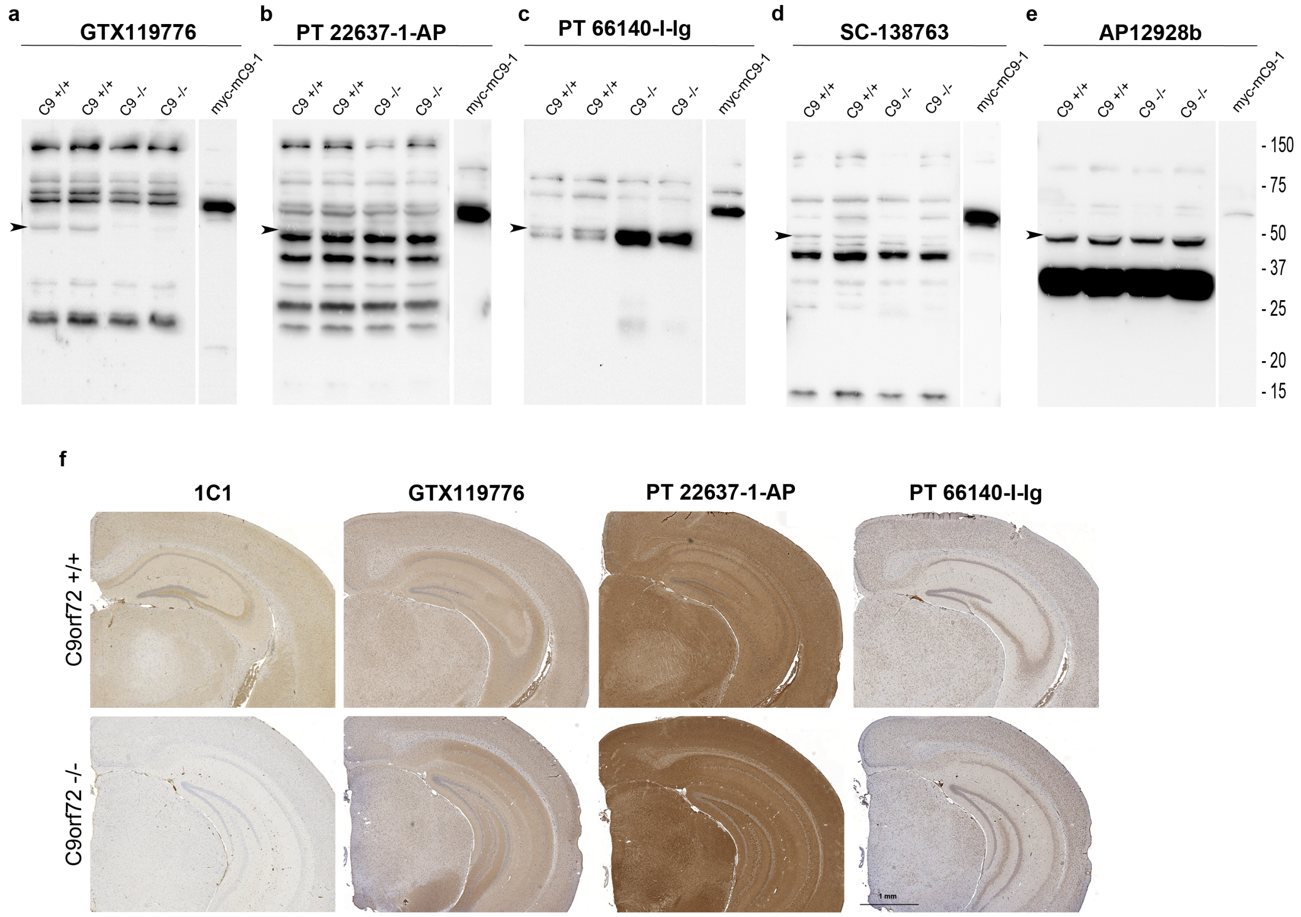

Figure S2. Commercially available C9orf72 antibodies tested on C9orf72 knock-out brain tissue. (a-e) Immunoblot analysis of total protein lysates from brains of wild-type ($C9^{+/+}$) and C9orf72 knock-out ($C9^{-/-}$) mice with different commercially available C9orf72 antibodies. Myc-tagged murine C9orf72 isoform 1 expressed in HEK293 cells is shown as control. (**a**) Genetex GTX119776, (**b**) Proteintech 22637-1-AP, (**c**) Proteintech 66140-I-Ig, (**d**) Santa Cruz-138763, (**e**) Abcam AP12928b. Arrowhead depicts potential C9orf72 band in brain lysates. Note that each antibody labels numerous unspecific bands. MW marker: Precision Plus Protein Dual Color Standards (**a-e**). (**f**) Immunohistochemistry of FFPE sections of wild-type ($C9^{+/+}$) and C9orf72 knock-out ($C9^{-/-}$) mouse brains stained with custom made C9orf72 antibody 1C1 (this study) compared to commercially available antibodies Genetex GTX119776, Proteintech 22637-1-AP and Proteintech 66140-I-Ig. Note that in contrast to 1C1, similar immunoreactivity is seen in wild-type and knock-out tissue with the tested commercially available antibodies as result of non-specific binding to unrelated proteins. Digital whole slide images were generated using a Zeiss Axioscan.Z1 digital slide scanner.

Figure S3

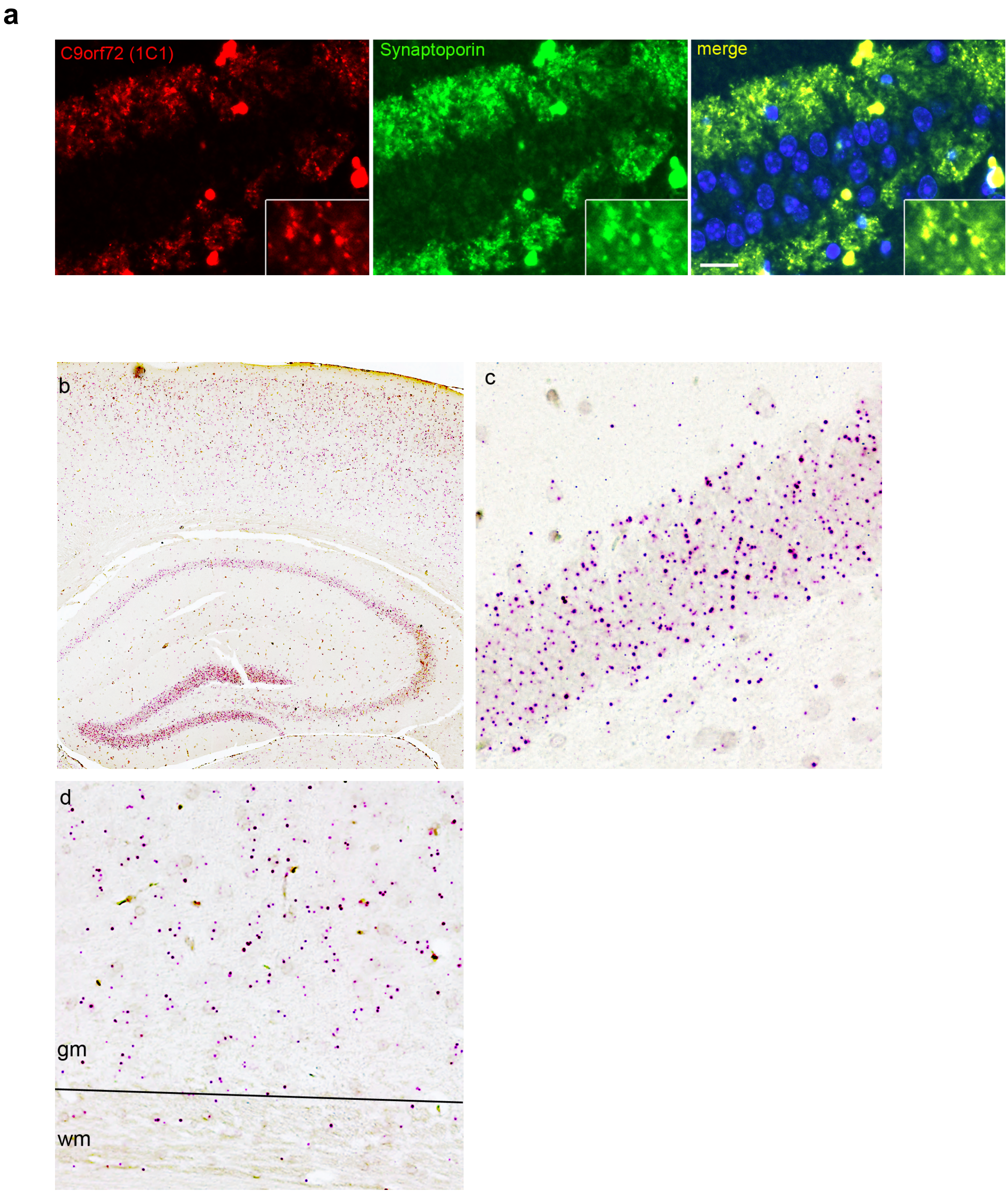

Figure S3: C9orf72 double-label immunofluorescence and C9orf72 *in situ* hybridization
(**a**) Partial co-localization of C9orf72 immunreactivity (red) was seen with the presynaptic protein marker synaptoporin (green) in the hippocampal mossy fiber system. Scale bar 20 µm and 6,5 µm for insert.
(**b**) Strongest C9orf72 mRNA expression is seen in the hippocampus and cortical gray matter in adult wild-type mice. Higher magnification of numerous cytoplasmic punctate dots in neurons of the dentate gyrus (**b**) and neurons in the frontal gray matter (gm) (**c**). Note that in the glial cells of the adjacent white matter (wm) only sparse dots are seen (**c**).

Figure S4

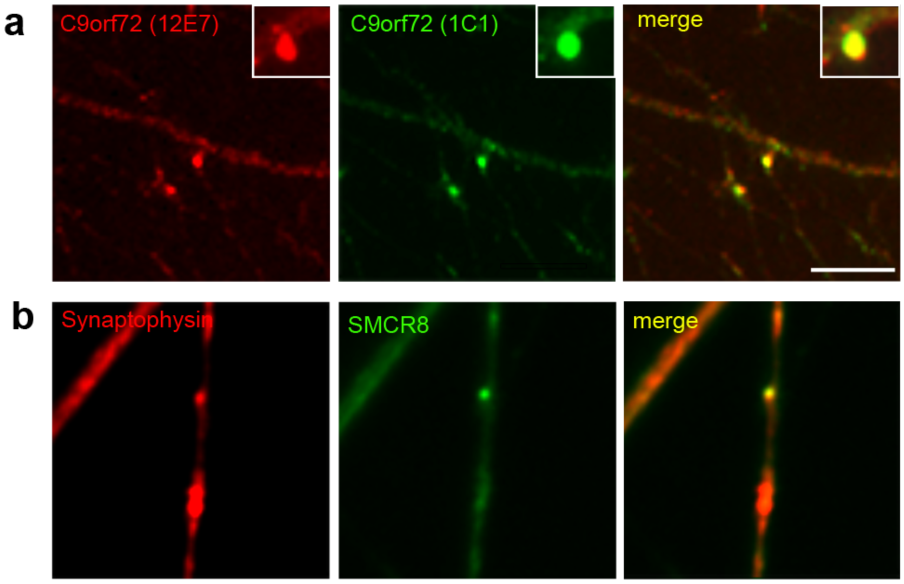

Figure S4: Immunofluorescence of human iPSC derived motor neurons
(**a**) Consistent co-localization of C9orf72 positive puncta is seen in 30 day old human iPSC derived motor neurons labeled with either rat monoclonal C9orf72 antibody 12E7 (green) or mouse monoclonal C9orf72 antibody 1C1 (red). (**b**) Synaptophysin-positive vesicles (red) partially co-localize with SMCR8 (green), similar to the partial co-localization observed between synaptophysin and C9orf72 shown in figure 4. Scale bar 1μm.

Figure S5

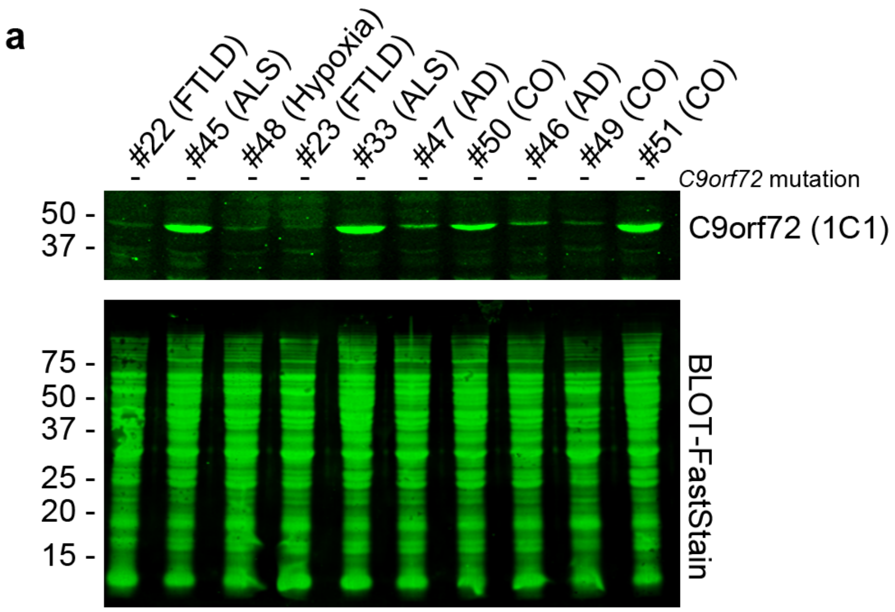

b

Case no	Degeneration/ cell death
#22	severe
#45	none
#48	severe
#23	severe
#33	none
#47	severe
#50	none
#46	moderate
#49	mild
#51	none

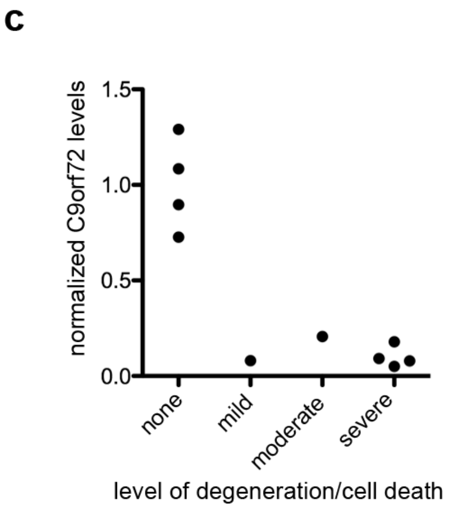

Figure S5. Immunoblot analysis of C9orf72 expression levels in frontal cortex
(**a**) Immunoblot for C9orf72 of RIPA lysates extracted from frozen frontal gray matter of cases without *C9orf72* mutation and with absence or presence of neurodegenerative changes/cell death. Total protein stains are shown as loading controls. AD, Alzheimer's disease; ALS, amyoytrophic lateral sclerosis; CO, neurologically healthy control; FTLD, frontotemporal lobar degeneration. MW marker: Precision Plus Protein Dual Color Standards. (**b**) Severity of neurodegeneration/cell death based on H&E stains. (**c**) Normalized C9orf72 protein levels show a strong negative correlation with levels of degeneration/cell death. Spearman rank correlation: rho = -0.834, p = 0.004.
